# AKT/mTOR as a targetable hub to overcome multimodal resistance to EGFR inhibitors in oesophageal squamous cell carcinoma

**DOI:** 10.1038/s41416-025-03093-3

**Published:** 2025-07-04

**Authors:** Lindsay C. Spender, Dale M. Watt, Mark A. Baxter, Morven K. Shuttleworth, Kateah Walker, Alice R. Savage, Hollie A. Clements, Yury Kapelyukh, Susan E. Bray, Sharon I. King, C. Roland Wolf, Gareth J. Inman, Shaun V. Walsh, Karen Blyth, Russell D. Petty

**Affiliations:** 1 Medical Oncology Group, Division of Cancer Research, Dundee, UK; 2 CRUK Scotland Institute, Glasgow, UK; 3 Division of Respiratory Medicine and Gastroenterology, Ninewells Hospital and School of Medicine, Dundee, UK; 4 Tayside Biorepository, Dundee, UK; 5 School of Cancer Sciences, College of Medical, Veterinary and Life Sciences, University of Glasgow, Glasgow, UK; 6 Department of Pathology, Ninewells Hospital and School of Medicine, Dundee, UK; 7 Present Address: Institute for Regeneration and Repair, University of Edinburgh, Edinburgh, UK; 8 Present Address: Molecular Cell and Developmental Biology, School of Life Sciences, University of Dundee, Dundee, UK

**Keywords:** Oesophageal cancer

## Abstract

**Background:**

Oesophageal squamous cell carcinoma (ESCC) is associated with late-stage diagnosis, limited treatment options, the development of drug resistance and poor outcome. Epidermal growth factor receptor is frequently dysregulated in ESCC. EGFR copy number gain and/or protein overexpression are beneficial as predictive biomarkers for EGFR inhibitor therapy; however, inherent and acquired resistance limit response rates, and durable disease control is infrequent.

**Methods:**

This study investigates the causes of resistance to the off-patent EGFR inhibitor gefitinib in three gefitinib-resistance model systems: intrinsic, acquired resistance and growth factor (TGFβ)-induced resistance. Findings from studies in 13 ESCC cell lines were validated in tumour specimens from the GO2 clinical trial (*n* = 32), publicly available ESCC datasets (*n* = 264), cell line-derived xenograft (CDX) and patient-derived organoid (PDO) model systems.

**Results:**

Gefitinib resistance in ESCC was associated with diverse mechanisms, including RTK signalling via PDGFRβ and IGFBP3/IGF1/IGF1R, as well as EMT, but was consistently associated with the maintenance of signalling via AKT across multiple cell lines and model systems. AKT or mTOR inhibitors synergised with gefitinib in 2D and anchorage-independent 3D assays. Gefitinib plus the AKT inhibitor capivasertib (Truqap™) was efficacious in human CDX and PDO models.

**Discussion:**

Combining AKT/mTOR inhibitors with EGFR inhibitors in EGFR-driven ESCC shows synergism but with elevated toxicity. Monotherapy AKT/mTOR inhibitors or combined therapy at reduced doses could offer improved, cost-effective therapy options for gefitinib-resistant cancer.

## Introduction

Oesophageal cancer is the sixth most common cause of death from cancer worldwide, with the dominant histological subtype being squamous cell carcinomas (ESCC) [1]. Neoadjuvant treatment and surgery are curative in fewer than half of patients, and, due to late-stage diagnosis, the majority of patients receive palliative cytotoxic chemotherapy [2]. Recent developments with immune checkpoint blockade (ICB) have improved treatment options in patients with advanced-stage ESCC, however, durable responses occur in a minority subgroup of patients [3] and five-year survival rates remain low at around 15%. There remains an unmet need for more effective treatments.

Efforts to characterise genomic aberrations in ESCC have identified the receptor tyrosine kinase epidermal growth factor receptor (EGFR) as a targetable oncogenic driver. Copy number gain (CNG) of *EGFR* analysed by FISH is detected in around 20% [4, 5] and EGFR is overexpressed in around 50% of tumours. When deregulated in ESCC, amplified EGFR signalling correlates significantly with tumour invasion [4], and is independently associated with poorer disease-free and overall survival [6]. Analyses of large-scale cancer dependency datasets demonstrate preferential strong EGFR dependency in ESCC [7].

EGFR can be targeted therapeutically using kinase inhibitors (TKi) (such as gefitinib) or monoclonal antibodies. Monotherapy trials in unselected patients indicate an EGFR-driven minority ESCC subgroup who gain survival, symptomatic control, and health-related quality of life benefits from EGFR inhibitors [8, 9]. Thus, patients whose tumours have EGFR CNG and/or EGFR protein overexpression represent a subgroup that potentially benefits from EGFR inhibitor monotherapy [5, 10], (HR for death, 0.21; 95% CI, 0.07 to 0.64; *P* = 0.006) [5]. However, even in this biomarker-selected subgroup, many patients either do not respond or do not have durable responses to EGFR-targeted therapy, indicating that primary intrinsic and acquired resistance are major clinical problems [11–13].

Aiming to improve efficacy, clinical trials in ESCC have investigated EGFR inhibitors when combined with standard cytotoxic chemotherapies, chemoradiotherapy or radiotherapy alone [14], with improvements in progression-free survival [15] and overall survival (OS) [16]. These trials underscored the importance of biomarker selection for the use of EGFR inhibitors in ESCC. In addition, the cytotoxic partner of gefitinib may also be important, for example, in vitro we have demonstrated antagonism with cisplatin or oxaliplatin, but synergy with docetaxel [17]. Optimisation of treatments, therefore, firstly to select the patients who are intrinsically sensitive to EGFR inhibitors and, secondly, to delay the outgrowth of drug-resistant tumours during EGFR targeted therapy, could provide better treatment options. This strategy has added clinical relevance, since, in ESCC, EGFR expression is inversely correlated with immune-cell infiltration, indicating that EGFR-driven ESCC is unlikely to respond well, or durably, to immune checkpoint inhibitor therapy (CPI) [18] as shown by the limited efficacy of CPIs in mutant-EGFR-driven NSCLC [19, 20]. Further caution is suggested by the observation that EGFR is associated with the concerning phenotype of CPI-induced hyper-progression, characterised by accelerated tumour growth and clinical deterioration [21].

To this aim, several studies have tried to identify mechanisms of acquired resistance that could be targeted in combination with EGFR inhibitors. Unlike NSCLC, escape mutation of EGFR in ESCC is uncommon, so treatment strategies are unlikely to be informed by targeting EGFR in NSCLC. Reactivation of MAPK or cyclin/cdk deregulation [13], epithelial-to-mesenchymal transition (EMT), SRC activation [12] and upregulation of other RTKs (AXL [13], FGFR [22], or c-MET [23] have all been implicated in resistance in ESCC, but there is no clear indication of which is the optimal target in the largest proportion of ESCC patients.

To address this, we studied both intrinsic and acquired resistance in ESCC to characterise potential biomarkers and mediators of diverse modes of resistance to an EGFR TKi and to identify improved drug therapy combinations. Gefitinib was selected for study as an off-patent, cost-effective EGFR inhibitor with proven benefit for biomarker-selected ESCC patients. We aimed to identify effective, approved or in-trial inhibitor combinations to enable more rapid progress from pre-clinical evaluation to the clinical setting to benefit a larger proportion of the EGFR CNG patient subgroup.

## Methods

### Cell lines

Human oesophageal squamous carcinoma cells (ESCC) derived from chemo-naïve patients have been described previously [24, 25]. Research resource identifier numbers (RRID) and culture protocols are detailed in Supplementary Methods.

### Patient-derived organoid (PDO)

The novel oesophageal squamous cell carcinoma patient-derived organoid A_DU_13997 was isolated in Matrigel from a 69-year-old female patient diagnosed with a poorly differentiated squamous cell carcinoma of the distal oesophagus and gastro-oesophageal junction using methods based on established protocols [26]. Tissue was collected with informed consent under Tayside Biorepository REC reference 22/ES/0041 - delegated approval TR726. Full details of protocols and immunohistochemistry validation are described in Supplementary Methods. PDO drug dose-response assays were carried out in 100 µL ESCC-specific organoid growth media with 2 × 10^3^ cells seeded in 5 µL Matrigel™. PDO growth was monitored in real time using an IncuCyte SX5 with an organoid software analysis module.

### Reagents and antibodies

Western blotting antibodies (with RRID numbers) and methods of analysis, human recombinant proteins (EGF, IGF-1, IGFBP3 and TGFβ), and the stock inhibitor solutions [AKT inhibitors: capivasertib (Truqap™, AZD5363) [27] and MK2206); mTOR inhibitors [INK128 (sapanisertib), everolimus (Afinitor®) and PP242 [28] used are described in Supplementary Methods.

### Knockdown with siRNA

Knockdown of gene expression of PIK3R1 p85α, PDGFRβ or IGFBP3 was carried out using 10 nM smart pool on-target plus siRNA (Dharmacon) with smart pool on-target plus non-targeting siRNA#2 as a control. Cells were transfected using Optimem and Lipofectamine RNAiMAX (Thermo Fisher Scientific) as recommended by the manufacturers.

### Gefitinib-resistant cell lines

Gefitinib-resistant TE11iresR#3, TE4iresR#2 and TE8iresR were generated and maintained in 2 µM gefitinib (Iressa) with the peak plasma concentration of gefitinib being 1–1.4 µM [29]. Matched control cell lines were generated to control for prolonged cell culture.

### Monitoring ESCC cell proliferation

ESCC drug dose-response analysis was monitored using adherent 2D and anchorage-independent 3D spheroid assays monitored by CellTiter-Glo® Luminescent Cell Viability Assay (Promega), crystal-violet colony formation assays or multiorganoid Incucyte analysis software. Assay protocols and analysis are detailed further in Supplementary Methods.

### Statistical analysis

IC_50_/EC_50_ and AUC values were determined from dose-response assays using CalcuSyn (Biosoft Version 2.0) (RRID:SCR_020251) or Graphpad prism software (RRID:SCR_002798) using non-linear regression analysis. Combination treatments were analysed by determining the drug combination index (CI) according to the Chou-Talalay method [30] using CalcuSyn software (CalcuSyn, Inc. Paramus, USA) which generates Dm values (IC50), dose-response curves and median effect plots. Recommended symbols for describing synergistic, additive, or antagonistic effects in drug combination studies analysed with the CI method (CalcuSyn user manual) are given where appropriate. Analysis of treatments versus control samples used GraphPad Prism one-way analysis of variance (ANOVA) with Dunnett’s post-test correction for multiple comparisons with one control sample. Superplots show all technical replicates within each of the biological replicate assays.

### Reverse phase protein array

The quantitative basal expression of 300 proteins and phospho-proteins commonly dysregulated in cancer was determined in three biological replicates of the ESCC cell line panel by the RPPA core facility, MD Anderson, Houston, Texas (RRID:SCR_016649). Further methods (in Supplementary Methods), and an antibody list is available on https://www.mdanderson.org/research/research-resources/core-facilities/functional-proteomics-rppa-core.html.

### Receptor tyrosine kinase array

Human proteome profiler phospho-receptor tyrosine kinase (RTK) arrays were used according to the manufacturer’s instructions (R&D Systems™). TE11 matched control and TE11iresR#3 cells were seeded and incubated overnight before cell lysis at 60-70% confluence. TE1 cells at 60–70% confluence were untreated or treated with 2 µM gefitinib for 4 h before harvesting in array lysis buffer. 200 µg lysate was used to probe the phospho-RTK array, and autoradiograph films were scanned after 5–10 min exposure.

### Scratch wound migration assay

Cells were seeded overnight at 2.5 × 10^4^/well in ImageLock 96-well plates (Essen Biosciences, Sartorius) and the monolayer serum-starved for 48 h before scratching using a Woundmaker (Essen Biosciences, Sartorius). Cells were either left untreated or were treated with serum-free media containing TGFβ1 (5 ng/mL), EGF (5 ng/mL) or IGF-1 (100 ng/mL). Cells were imaged every 2 h using an IncuCyte Zoom™ (Essen Bioscience), and the mean ± sem relative wound density was analysed by IncuCyte 2018A software from 6 wells/1 image/well.

### Analysis of datasets

#### GO2 trial data analysis

Details of the GO2 clinical trial [31] RNA extraction and sequencing, which included 32 squamous cell carcinoma patients, are detailed in Supplementary Methods. Biospecimen collection (ethical approval REC Number 13/YH/0229) was conducted by NHS Grampian Biorepository (16/NS/0055) and registered with the NHS Tayside Biorepository (REC approval 17/ES/0130). RNA sequencing data analysed for associations between expression groups and progression-free and overall survival were investigated using R statistical software (version 4.0.2).

#### Publicly accessible dataset analysis

Kaplan-Meier ESCC patient overall survival curves, grouped according to ESCC tumour RNA expression levels, were generated using the online survival tool OSescc (accessed March 2025) [32] from clinical and RNAseq datasets of 264 ESCC patients compiled from GSE53625 (*n* = 179) [33] (Gene Expression Omnibus (GEO; ncbi.nlm.nih.gov/geo/)) and The Cancer Genome Atlas (TCGA; cancergenome.nih.gov) (*n* = 85). Overall and disease-specific survival (*n* = 78 ESCC patients) according to median tumour RPPA protein expression data for pIGF1R (pY1135/1136) was analysed using TRGAted (https://nborcherding.shinyapps.io/TRGAted/) [34].

### Cell line-derived murine xenograft (CDX) study

#### Subcutaneous transplants

TE-4 xenografts (5 × 10^6^ cells injected subcutaneously into 16-18-week-old female BALB/c Nude mice (Charles River, UK)) were performed following UK Home Office regulations, under project license PP6345023, with approval from the Animal Welfare and Ethical Review Board of the University of Glasgow. Details of the in vivo drug treatments on day 18 post-transplant are given in Supplementary Methods.

## Results

### Characterisation of the intrinsic gefitinib-response phenotype in ESCC cell lines

**Fig. 1:**
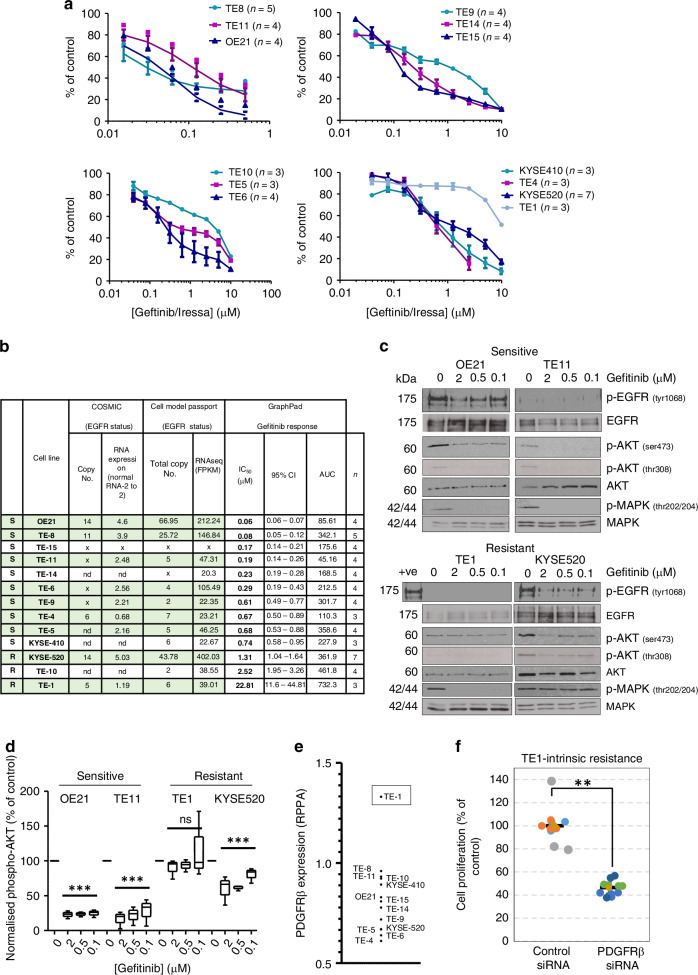
Profiling ESCC cell line sensitivity to gefitinib. **a** 13 ESCC cell lines were profiled by CellTitre-glo® luminescent cell viability assay for their sensitivity to gefitinib. Data shown are non-linear curve fits, mean ± s.d. percent cell viability relative to untreated control cells (100%) from replicate (n) independent assays. **b** ESCC cell lines: EGFR copy number and RNA expression status obtained from COSMIC cell lines project (https://cancer.sanger.ac.uk/cell_lines) (data derived from TCGA or ICGC) and Cell Models Passport (https://cellmodelpassports.sanger.ac.uk/). https://depmap.sanger.ac.uk/documentation/datasets/copy-number/. x = no copy number variation or expression recorded. Gefitinib sensitivity (IC_50_, 95% CI and area under the curve, AUC) were determined by GraphPad Prism from independent replicate assays (n) shown in (**a**). Cell lines with dysregulated EGFR (either CNG and/or RNA expression) confirmed in both the COSMIC and Cell Models Passport databases and are highlighted in colour. **c**, **d** Intrinsically gefitinib-sensitive (OE21 and TE-11) and resistant (TE-1 and KYSE-520) ESCC cells were treated for five hours with solvent control (0) or gefitinib (0.1, 0.5, 2 µM). Equal amounts of protein cell lysates were analysed by SDS-PAGE and western blotting. **d** Quantification of western blots (shown in **c**); normalised phospho-AKT levels (p-AKT ser 473/pan AKT) following gefitinib treatment (box-and-whiskers min to max plot from *n* = 3 independent experiments). Statistical analysis was performed in GraphPad Prism (Dunnett’s multiple comparison test). **e** Normalised PDGFRβ protein expression in the panel of ESCC cell lines determined by RPPA. **f** Superplot of CellTitre-glo® viability assays following transfection of non-targeting and PDGFRβ targeting siRNA in TE-1 cells independent experiments (*n* = 3) with the technical replicates within each assay shown. Statistical analysis was performed in GraphPad Prism (Student’s TTEST) using the means of independent assays.

To identify mediators of resistance to EGFR inhibition, the intrinsic gefitinib sensitivity of a panel of 13 well-characterised ESCC cell lines was determined by dose-response viability screens (Fig. 1a, b). Cell lines with EGFR dysregulation, confirmed by both COSMIC and Cell Model Passport databases, are highlighted in Fig. 1b in colour. The cells exhibited a range of responses to gefitinib; ten were relatively sensitive (S) while three lines were more intrinsically resistant (R) with an IC50 at or above the peak plasma concentrations of gefitinib (1–1.4 µM). These responses modelled observed clinical trial responses of patients to EGFR inhibitors, highlighting current limitations of biomarker selection with primary intrinsic resistance evident despite the presence of the EGFR CNG biomarker (eg TE-1).

To identify which cancer-associated proteins correlated with gefitinib sensitivity, we performed a reverse-phase protein array (RPPA) with regression analyses using protein expression data plotted against gefitinib IC50 values. The regulatory subunit of the PI3K signalling pathway, p85α, encoded by *PIK3R1*, was among the hits whose expression significantly correlated with the gefitinib IC50 of the cell lines (*p* = 0.0002, R^2^ 0.73), with low expression in the most resistant lines (Figure S1A). Western blots confirmed the inverse correlation (Figure S1B). These data implicate the PI3-Kinase/AKT pathway in determining gefitinib sensitivity.

### Maintenance of AKT signalling is associated with intrinsic and acquired gefitinib-resistance in ESCC cell lines

We investigated PI3K/AKT signalling in response to gefitinib in cell lines with confirmed EGFR dysregulation to model those patients likely to receive EGFR TKi treatment: two sensitive (lowest IC50 and AUC values) (TE-11 and OE21) and two resistant cell lines (TE-1 and KYSE-520). Following gefitinib treatment, there was more inhibition of phospho-AKT in intrinsically sensitive cells than in gefitinib-resistant cells shown by western blot (Fig. 1c) and quantified (Fig. 1d). Efficient knockdown of PIK3R1 p85α in gefitinib-sensitive cells (Figure S2A) did not reduce sensitivity (Figure S2B), suggesting that loss of PIK3R1 p85α is unlikely to be directly causative but may be a marker of gefitinib resistance.

The RPPA data also revealed that expression of the RTK platelet-derived growth factor beta (PDGFRβ) correlated significantly with resistance (linear regression analysis; *p* = 0.03) (Figure S3A), with the highest expression seen in resistant TE-1 cells (Fig. 1e, boxed). Proteomic profiling using a phospho-RTK array confirmed the expression of active phosphorylated PDGFRβ (Figure S3B), which, apart from detectable active EGFR and HER3, was the only readily detectable active RTK expressed in TE-1 cells. PDGFRβ inhibition (Figure S3C) and knockdown (Figure S3D) reduced TE-1 proliferation (Figure S3C and Fig. 1f, respectively); thus, EGFR CNG^+ve^/EGFR protein^low^ TE-1 cells depend largely on the alternative RTK PDGFRβ for growth.

To investigate whether maintenance of AKT signalling was associated more generally with gefitinib resistance, two additional model systems were used, acquired resistance and growth factor (TGF-β)-induced resistance.

**Fig. 2:**
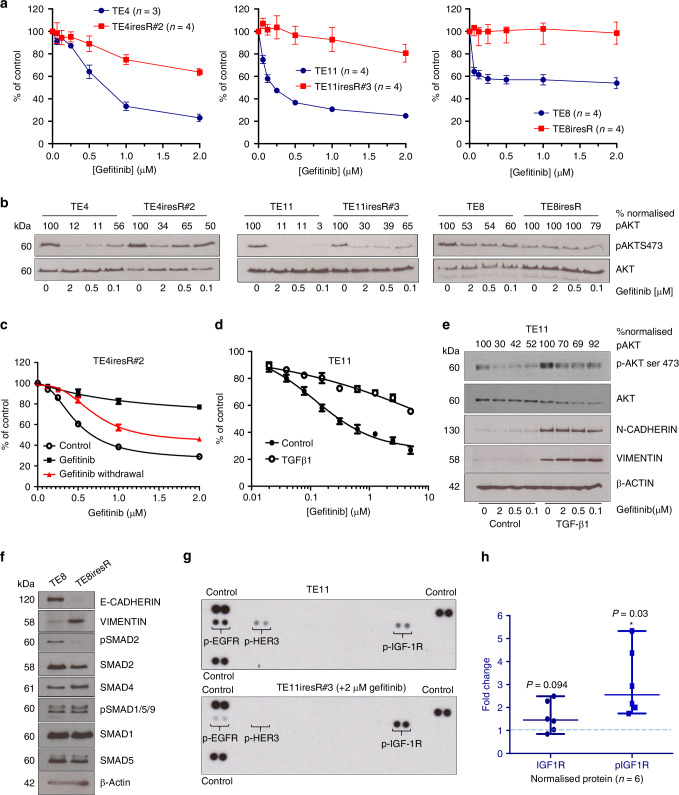
Multiple modes of gefitinib resistance are associated with the maintenance of phospho-AKT expression. **a** Acquired gefitinib-resistant and matched control cell lines were analysed by adherent CellTitre-Glo® viability assays and are shown as GraphPad Prism dose-response curves (mean ± sem) from replicate biological assays (*n* = ≥3). **b** Gefitinib-resistant and matched control cell lines [shown in (**a**)] were treated for five hours with solvent control (0) or gefitinib (0.1, 0.5, 2 µM). Equal amounts of protein lysates were analysed by SDS-PAGE and western blot. Percent normalised p-AKT levels (p-AKT ser 473/pan AKT) were quantified using Image Studio relative to untreated controls. **c** TE4iresR cells were maintained without gefitinib selection media for between 4 and 8 weeks (gefitinib withdrawal) before comparing gefitinib dose-response curves (5000 cell/well) with parental TE-4 cells (control). Data presented are non-linear regression curve fits analysed in GraphPad prism from independent experiments (*n* = 3). **d** TE-11 ESCC cells were left untreated or pre-treated during routine passage with 5 ng/mL TGF-β1 for 2 weeks and then analysed by CellTitre-Glo® viability assay for sensitivity to gefitinib. Data shown are GraphPad Prism non-linear regression curves from biological replicate assays with six replicate wells per assay (*n* = 2). **e** TGF-β1 pre-treated and matched control cell lines [shown in (**d**)] were treated for five hours with solvent control or gefitinib (0.1, 0.5, 2 µM) and equal amounts of lysates analysed by SDS-PAGE and western blot. A β-actin western is included as a sample integrity control. **f** TE-8 matched parental and TE8iresR protein lysates were analysed by SDS-PAGE and western blot. β-actin is included as a loading control (for the vimentin blot) or sample integrity blot. **g** TE-11 and TE11iresR#3 cells were seeded in 10 cm dishes for 24 h before lysing and 200 µg protein analysed by proteome profiler phospho-receptor tyrosine kinase array (R&D systems). **h** Image Studio software quantification of western blots for IGF1R and phospho-IGF1Rβ tyr1135 in TE11 and TE11iresR#3. Normalised (loading control) ratios of protein expression levels in TE11iresR#3 cells compared to TE11 control were analysed by Wilcoxon signed rank test from replicate experiments (*n* = 6) using Graphpad Prism; Wilcoxon ns not significant (*p* = 0.094), *p = 0.03)

We induced acquired resistance by prolonged exposure to gefitinib in three sensitive cell lines (TE4iresR#2, TE8iresR and TE11iresR#3) (Fig. 2a). TE4 was selected as an example of cells with confirmed CNG and normal EGFR expression, while TE-8 and TE-11 cells were chosen as examples of cells with CNG and protein overexpression. Although OE21 cells were the most sensitive, they were not selected because they have been well-characterised in other resistance studies [13].

Note that, unlike gefitinib-resistant ESCC cell lines derived previously, which were generated by exposure to increasing incremental doses of EGFR TKi [13], our panel of resistant lines were generated by the addition of just above (2 µM gefitinib) peak plasma levels (1–1.4 µM) of gefitinib from the outset. We believed that our approach could more likely replicate the selective pressures and generate the mechanisms of resistance seen in tumours since clinical chemotherapy regimens are not based on incremental increases in drug dose but on fixed doses according to body weight.

Like intrinsic resistance, we found that acquired resistance was associated with maintained phospho-AKT (Fig. 2b). Resistance could be partially reversed by drug withdrawal (Fig. 2c).

In the third resistance model (growth factor-induced), pre-treatment of TE-11 cells with TGF-β1 also promoted drug resistance (Fig. 2d) and maintenance of phospho-AKT (Fig. 2e). We considered whether epithelial-to-mesenchymal transition (EMT) was induced which is frequently caused by TGFβ1 signalling and has been linked previously to gefitinib resistance phenotypes in ESCC [35]. As predicted, in addition to the maintenance of p-AKT, TGFβ1 induced the EMT markers N-CADHERIN and VIMENTIN (Fig. 2e). The acquired-resistant cell line TE8iresR also showed clear evidence of EMT (Fig. 2f), which was associated with lower expression of the TGF-β signalling mediators SMAD2 and phospho-SMAD2, which may indicate an alteration in TGF-β canonical signalling. However, in contrast, we saw no evidence of EMT in TE4iresR cells and only a partial EMT (increased N-CADHERIN, but no change in VIMENTIN or E-CADHERIN) in TE11iresR cells (Figure S4). EMT, therefore, does not account for the gefitinib resistance phenotypes in all model systems, which is consistent with afatinib-resistant ESCC cells [12].

Maintained AKT activation was therefore considered the most consistent correlate with resistance across multiple cell lines and in all resistance models.

### IGFBP3/IGF-1/IGF1R axis mediates EGFR inhibitor resistance via AKT/mTOR signalling in ESCC

Given that intrinsically resistant TE-1 cells depend on an alternative RTK for growth despite having EGR CNG, and given previous observations in other studies, we tested whether bypass signalling via other RTKs was the predominant mediator of maintained signalling through AKT in our acquired-resistance model. We profiled phospho-receptor tyrosine kinases in TE11iresR#3 resistant cells compared with their parental controls. Phospho-EGFR and phospho-HER3 were decreased, showing target inhibition, and we noted slight increased expression of insulin-like growth factor 1 receptor IGF1R and significantly increased (*p* = 0.03) phosphorylation of IGF1R (Fig. 2g, h), which was confirmed by western blotting (Figure S5A).

**Fig. 3:**
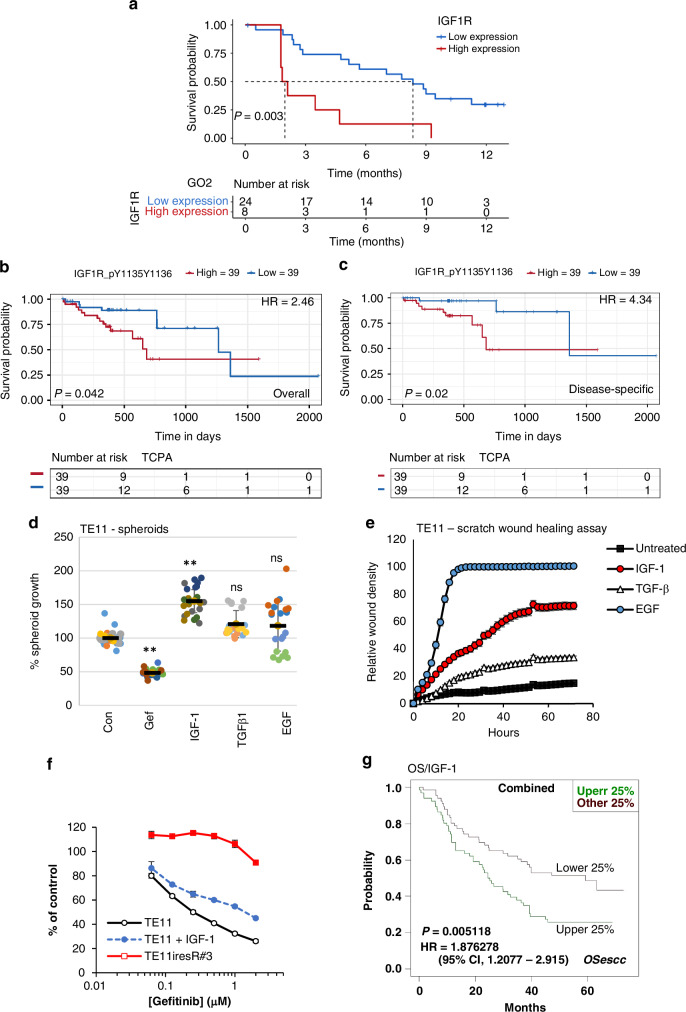
IGF1R signalling in ESCC is associated with poor prognosis, resistance to gefitinib, spheroid growth and migration of ESCC cells. **a** Overall survival (OS) in the GO2 trial ESCC population according to *IGF1R* RNA expression. High expression was defined as the top 25% of expressors. Low expression was defined as the bottom 75% of expressors. **b**, **c** TCGA tumor RPPA data from ESCC patients (*n* = 78) were analysed using TRGAted (accessed 07/2024). Overall (**b**) (HR, 2.46; *p* = 0.042) and disease-specific (**c**) (HR, 4.34; *p* = 0.02,) survival in TCGA dataset patients with median high and low expression of pIGF1R (pY1135/1136). **d** TE-11 spheroids in poly-HEMA coated round-bottomed plates were treated with media alone (control), or media supplemented with Gefitinib (2 µM), EGF (5 ng/mL), TGFβ1 (5 ng/mL) or IGF-1 (100 ng/mL) for 5 days. Viable cells were assayed by CellTitre-glo® and the results presented as a SuperPlot of six data points obtained from each replicate biological assay (*n* = 4). Statistical analysis of treatments relative to control samples was conducted using GraphPad Prism software (ANOVA with Dunnett’s correction, **p ≤ 0.01, ns = not significant). **e** Migration scratch assay; TE-11 cells were seeded at 25,000/well overnight in ImageLock plates and cell monolayers serum-starved (0% serum) for 48 h before wounding. Cells were treated with serum-free media (control), or serum-free media plus EGF (5 ng/mL), TGFβ1 (5 ng/mL) or IGF-1 (100 ng/mL) and analysed by IncuCyte Zoom real-time imaging. The relative wound closure ± sem of six replicate wells in a representative experiment is shown. **f** TE-11 cells were seeded with and without exogenous IGF-1 (100 ng/mL) and analysed in gefitinib sensitivity assays in comparison with TE11iresR#3 cells. Cell viability (mean % of control ± s.d) was assessed by CellTitre-glo® (**g**) OSescc overall survival analysis of ESCC patients with upper and lower quartile *IGF-1* RNA expression (combined data from TCGA and GSE53625).

Analysis of public datasets of oesophageal cancer RNA expression revealed that *IGF1R* RNA was elevated in tumours compared to normal tissue (Figure S5B). In western older, frailer advanced ESCC patients in the GO2 clinical trial (*n* = 32), worse median overall survival was observed in the high versus low *IGF1R* expression group [2.0 months (95% CI; 1.8–NA) versus 8.3 months (5.2–NA) (HR, 3.99; 95% CI 1.62–9.80, *p* = 0.003)], respectively (Fig. 3a). The poorer prognosis of patients with high *IGF1R* expression was confirmed in an independent dataset (Figure S5C). Interestingly, *IGF1R* was elevated in ESCC compared with EAC patients (*p* = 0.0048) within the GO2 cohort (Figure S5D), and we saw no significant effect of *IGF1R* expression on OS in the EAC population (Figure S5E), suggesting that IGF1R may be a poor prognostic factor selectively in squamous carcinoma.

Analysing the correlation between active phospho-IGF1R protein expression and prognosis using the TCGA TRGated RPPA dataset of ESCC tumour tissue, high median expression of phospho-IGF1R (pY1135/pY1136) was significantly correlated with both poorer OS (*p* = 0.042, HR, 2.46) (Fig. 3b) and disease-specific survival (*p* = 0.02, HR, 4.34) (Fig. 3c) of ESCC patients (n = 78) while no significant difference was observed in EAC (n = 48) (data not shown).

Since IGF1R activation in ESCC is associated with poor prognosis, we investigated its function in cell lines. IGF1Rβ receptor subunit was expressed across the cell line panel, but levels did not correlate with intrinsic sensitivity to gefitinib (Figure S5F). 3D anchorage-independent spheroid growth (Figure S6A, Fig. 3d) and migration assays (Figure S6B, Fig. 3e) in gefitinib-sensitive TE-11 cells showed that the IGF1R ligand IGF-1, unlike EGF and TGFβ1, significantly increased spheroid growth and was also more efficient than TGFβ1 in promoting ESCC cell migration. These data extend and support previously published ESCC cell line data, where IGF-1 promoted migration in TE-1 cells [36].

In drug sensitivity assays, the addition of IGF-1 to TE-11 cells partially reduced sensitivity to gefitinib (Fig. 3f), which established a causal link between induced IGF1R signalling and drug resistance. In ESCC patients, tumour expression of *IGF-1* RNA in the top versus the bottom quartile expression groups was associated with worse overall survival (OS) (HR, 1.876; 95% CI 1.2007 – 2.915, *p* = 0.005) (Fig. 3g).

**Fig. 4:**
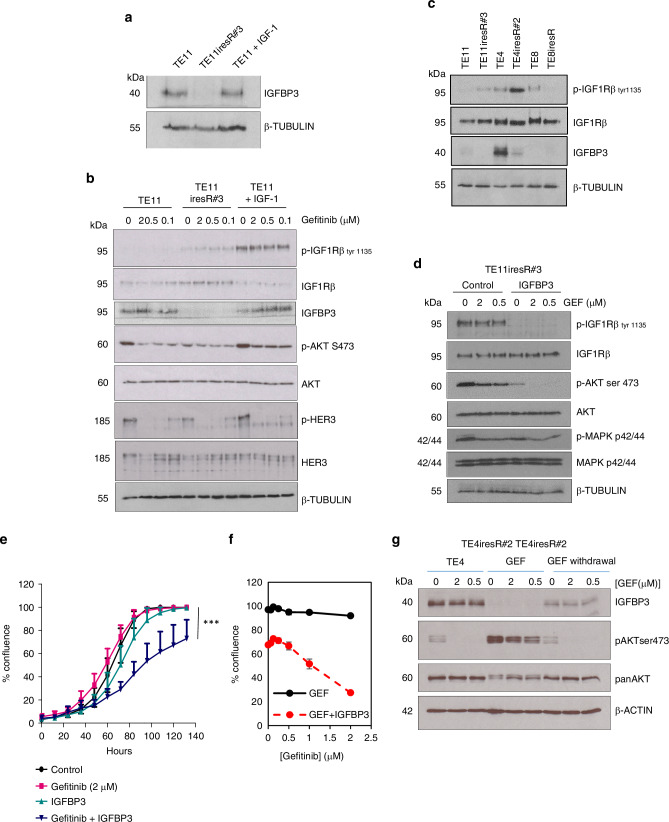
IGFBP3 downregulation is associated with elevated IGF1R signalling and resistance to gefitinib. **a** TE-11 cells were treated with and without exogenous IGF-1 (100 ng/mL) and analysed by SDS-PAGE and western blotting in comparison with TE11iresR#3 cells. A β-TUBULIN western is included as a sample integrity control. **b** TE11 cells treated as in (**a**) were then treated for 5 h with solvent control, 0.1, 0.5 or 2 µM gefitinib before analysis by SDS-PAGE and western blotting. β-TUBULIN western is included as a sample integrity control. **c** TE11, TE4 and TE8 gefitinib-resistant cell lysates and matched control cell lines were analysed by SDS-PAGE and western blotting. β-TUBULIN is shown as a sample integrity control and loading control for IGF1Rβ (**d**) TE11iresR#3 cells were seeded overnight and pre-treated with BSA diluent control or 2.5 µg/mL IGFBP3 for 1 hr before a five-hour treatment with gefitinib (0, 0.5, 2 µM). Protein lysates were prepared and analysed by SDS-PAGE and western blotting. β-TUBULIN is included as a sample integrity control and loading control for IGF1Rβ. **e** TE11iresR#3 cells were seeded overnight in 96-well plates before treatment with solvent control, gefitinib (2 µM), IGFBP3 (2.5 µg/mL) or gefitinib plus IGFBP3. Cell proliferation (mean percent confluence) was analysed by real-time IncuCyte imaging. Results from independent assays (*n* = 3) were analysed using one-way analysis of variance (ANOVA) with Dunnett’s post-test correction for multiple comparisons using GraphPad prism. (*** = *p* ≤ 0.001). **f** TE11iresR#3 cells were treated for four days with a gefitinib with and without the addition of 2.5 µg/mL IGFBP3. Cell proliferation (mean per cent confluence) was analysed by real-time IncuCyte imaging (*n* = 3). **g** TE4iresR#2 cells, usually maintained in 2 µM gefitinib (GEF), were subjected to a drug holiday by removal of gefitinib (as described in Fig. 2C (GEF withdrawal). Lysates from gefitinib-treated cells (5 hours) were analysed by SDS-PAGE and western blotting. β-ACTIN is shown as a sample integrity control.

IGF-1 signalling via IGF1R is regulated by IGF-binding proteins, which sequester ligands. Loss of expression of the IGFBPs, IGFBP3 and IGFBP4, has previously been linked with resistance to gefitinib in other cancer types [37] and IGFBP-3 is frequently concomitantly overexpressed with EGFR in ESCC [38]. We therefore compared IGFBP3 expression in gefitinib-resistant and parental control cells and discovered that IGFBP3 was downregulated in acquired resistance and its levels were unaffected by the exogenous addition of IGF-1 to parental TE-11 cells (Fig. 4a). Because exogenous IGF-1 only partially induced gefitinib resistance in TE-11, we checked whether IGF1R activation by IGF-1 was limited by the higher expression of endogenous IGFBP3 found in TE-11 compared to TE-11iresR#3 cells. Exogenous IGF1 induced p-IGF1R and p-AKT above the basal levels seen in TE-11 and TE-11iresR#3 cells (Fig. 4b), and so did not appear limiting.

Importantly, changes in pIGF1R and IGFBP3 occurred in two distinct cell backgrounds, TE11iresR#3 and TE4iresR#2 (Fig. 4c), so this could be a more general resistance mechanism. To test whether the loss of IGFBP3 determined gefitinib resistance, recombinant IGFBP3 was added to TE11iresR#3 cells before treatment with gefitinib. Exogenous IGFBP3 reduced basal pIGF1R and pAKT, confirming that AKT is activated downstream of IGF1R in gefitinib-resistant ESCC, and rescued inhibition of pAKT (Fig. 4d) and significantly resensitised TE11iresR#3 cells to gefitinib (Fig. 4e, f). The gefitinib drug holiday experiments showing gefitinib resistance reversal (Fig. 2c) were associated with a rebound of IGFBP3 expression and inhibition of phospho-AKT by gefitinib (Fig. 4g). An IGF1R inhibitor (PQ401) reduced ESCC cell proliferation (Figure S7A and S7B) and synergised with gefitinib in TE-11 cells (Figure S7C), which is consistent with data using different ESCC cell lines and inhibitors [39]. However, transient IGFBP3 knockdown using siRNA (Figure S7D) was not sufficient to induce gefitinib resistance (Figure S7E). We conclude, therefore, that the IGF-1/IGFBP3/IGF1R axis can regulate acquired gefitinib resistance but that other factors may also be involved.

### AKT/mTOR inhibition overcomes multimodal resistance to EGFR inhibitors in ESCC

**Fig. 5:**
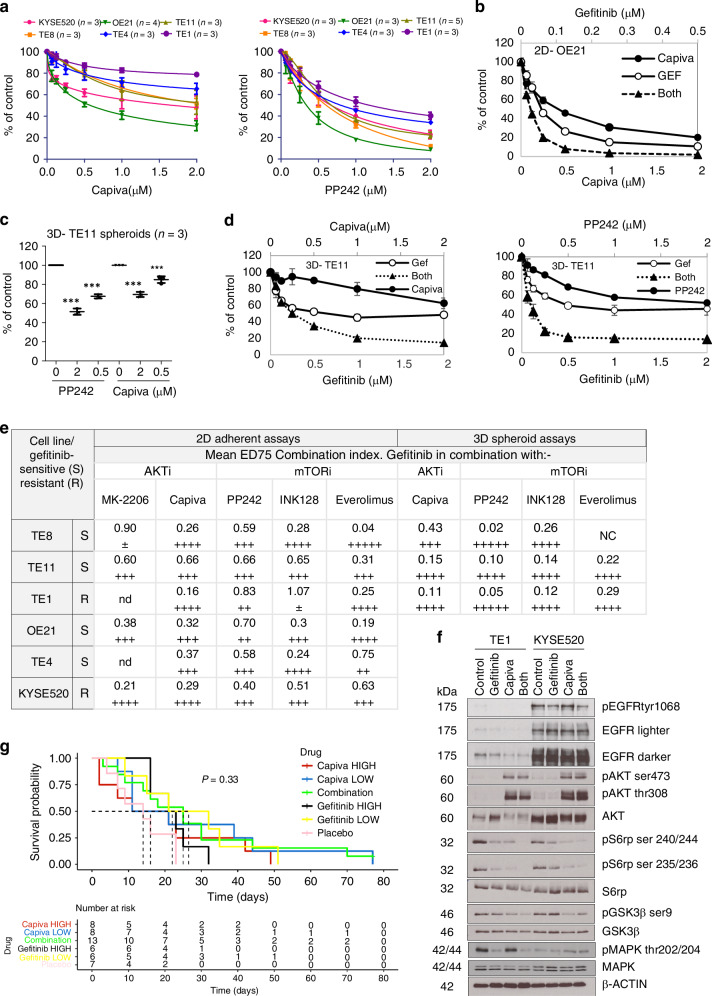
Targeting AKT or mTOR acts synergistically with gefitinib. **a** Monotherapy AKT inhibitor (Capivasertib) and mTOR inhibitor (PP242) dose-response curves in intrinsically gefitinib-sensitive (OE21, TE-11, TE-8 and TE-4) and gefitinib-resistant (KYSE-520 and TE-1) ESCC cells determined by CellTitre-glo® viability assays. **b** Representative 2D- adherent drug combination dose-response curves showing gefitinib (GEF) or capivasertib alone and in combination (Both). **c** Effect of 5 days capivasertib and PP242 treatment on TE-11 spheroid growth. Data shown are CellTitre-glo® viability counts as a percentage of control [mean ± s.d. of independent experiments (*n* = 3)]. **d** Representative 3D-TE-11 spheroid drug combination assays. Cells were treated with gefitinib alone or gefitinib in combination with PP242 or capivasertib. **e** Summary table of mean ED75 combination indices (CI) of gefitinib combined with AKT inhibitors (AKTi) or mTOR inhibitors (mTORi) in ESCC cell lines with EGFR dysregulation determined from at least two adherent and spheroid assays each with 3 replicate wells (as shown in **b**, **d**). CI < 1 = synergy, >1= antagonism; the degree of synergy is given as: +++++ = very strong synergism, ++++ = strong synergism, +++ = synergism, ++ = moderate synergism, ± = nearly additive. 3D spheroids of KYSE520, OE21 and TE-4 were not viable. NC = could not be accurately calculated by Chou-Talalay modelling. nd = not done. **f** Western blot analysis of intrinsically resistant cell lines TE-1 and KYSE-520 following 24 h of treatment with gefitinib (2 µM), capivasertib (0.5 µM) or both drugs. β-ACTIN is included as a sample integrity control. **g** Survival analysis of mice in CDX experiment following treatment of xenografts with gefitinib: 100 mg/kg (high), 50 mg/kg (low), capivasertib: 130 mg/kg (high), 85 mg/kg (low) or combination low doses gefitinib (50 mg/kg) + capivasertib 85 mg/kg), 4 days on/3 days off).

Targeting IGF1R may have utility in preventing gefitinib-resistant disease, but IGF1R inhibitors have previously shown variable response rates and unacceptable toxicity in clinical trials [40]. The diversity of drug resistance mechanisms would also make combination drug selection challenging in the real-world clinical setting. An alternative, to widen patient population groups likely to benefit, would be to target the convergent downstream AKT or mTOR effectors of these different mechanisms, an approach also benefiting from several inhibitors already being in clinical development or use. Three small molecule inhibitors were therefore selected for testing in ESCC cells in the first instance, targeting AKT [MK-2206 and capivasertib (AZD5363)] or mTORC1/2 (PP242) (Figure S8A). The allosteric AKT inhibitor MK-2206 has previously been reported to inhibit proliferation of several of the KYSE cell line series [41], but we now show its effects are cell line dependent, with TE11 cells being unaffected (Figure S8B). As single agents, the active site mTOR inhibitor PP242 inhibited ESCC growth more effectively than capivasertib targeting AKT (Fig. 5a). In migration assays, capivasertib also had no effect, but the mTOR inhibitor PP242 partially inhibited EGF-induced migration (Figure S8C). However, gefitinib combined with capivasertib or PP242 showed improved inhibition of cell proliferation in 2D adherent assays in both intrinsically sensitive and resistant cells (a representative example is shown in Fig. 5b). Likewise, capivasertib and PP242 as monotherapies only partially inhibited the growth of TE-11 cells in 3D spheroid assays (Fig. 5c) but were highly effective in combination with gefitinib in the two cell lines that were able to form spheroids (TE-8 and TE-11) (examples shown in Fig. 5d). We extended our study to test two more mTOR inhibitors currently in clinical trials or approved for use, the active site inhibitor sapanisertib (INK-128) and the selective mTORC1 inhibitor everolimus, which binds FKBP12. Everolimus has previously been reported to inhibit phosphorylation of mTOR substrates and ESCC cell growth and to induce apoptosis in ESCC cell lines at the concentrations used here [42]. While everolimus monotherapy has not progressed for the treatment of gastrointestinal tumours, it may have utility in combination therapies. Drug combination assays across the cell line and drug panel were analysed by calculating Chou-Talalay Combination indices (CI); the results are summarised in Fig. 5e. Like capivasertib and PP242, INK-128 and everolimus acted synergistically with gefitinib (CI < 1). To assess the possible mechanism of improved efficacy of the combination therapy, we analysed kinetic cell proliferation by real-time IncuCyte imaging (Figure S9) and signalling via the AKT/mTOR pathway by western blot at 24 h post-treatment (Fig. 5f). Doses of gefitinib (2 µM) and capivasertib (0.5 µM) had marginal effects as monotherapies but effectively reduced cell number when used in combination (Figure S9). Capivasertib alone induced feedback ser 473 and thr 308 AKT phosphorylation as reported previously but inhibited phosphorylation of the downstream target S6 ribosomal protein at ser 235/236 and ser 240/244 (Fig. 5f). More inhibition of pS6rp occurred in both gefitinib-resistant cell lines when gefitinib and capivasertib were combined. We conclude that combination therapy is a more effective inhibitor of mTOR signalling and provides supportive data for the synergistic activity of gefitinib with mTOR inhibitors.

We selected the AKT inhibitor capivasertib for further studies in a CDX model based on its synergy profiles, its availability and its FDA approval for clinical use (in breast cancer). Three cell lines were piloted for xenograft formation (TE-8, TE-11 and TE-4) and, of these, TE-4 cells were selected because of superior xenograft formation and confirmed synergistic response to gefitinib + capivasertib (Figure S10). The combination dosages and schedules were reduced from the standard monotherapy doses of 100 mg/kg for gefitinib and 130 mg/kg capivasertib to limit toxicity; combination doses were gefitinib (50 mg/kg) + capivasertib 85 mg/kg), 4 days on/3 days off. Our experiments were complicated by the aggressive nature and rapid ulceration of the xenografts, which necessitated early harvesting of some mice (Table S2). Nevertheless, Cox regression analysis showed significant improvement in survival with the combination (HR, 0.33; 95% CI, 0.12–0.88; *p* = 0.028) compared with standard doses of Gefitinib (100 mg/kg) (HR, 0.51; 95% CI, 0.17–1.52; *p* = 0.2) and capivasertib (130 mg/kg) (HR, 0.54; 95% CI, 0.19–1.55; *p* = 0.3) (Fig. 5g and Table S3). Unexpectedly, we also found slightly improved survival with lower dose monotherapy; Gefitinib (50 mg/kg) (HR, 0.36; 95% CI, 0.12–1.12; *p* = 0.078) and capivasertib (85 mg/kg) (HR, 0.54; 95% CI, 0.19–1.55; *p* = 0.3) (Table S3), the reason for this is unclear. Drug combinations targeting EGFR and the downstream effectors AKT or mTOR were therefore more effective than monotherapy but are associated with increased toxicity in vivo.

Since the development of acquired resistance remains a major problem for the clinical use of EGFR inhibitors, we investigated whether AKT and mTOR inhibitors could resensitise gefitinib-resistant lines. We tested gefitinib titrations in the presence of fixed concentrations of capivasertib (2 µM) and PP242 (0.2 µM) that produced a modest reduction in cell proliferation as single agents (Figure S11A). Capivasertib (2 µM) rendered two lines, TE4iresR#2 and TE11iresR#3, more sensitive to gefitinib. PP242 (0.2 µM) rendered TE11iresR#3 more sensitive to gefitinib (Figure S11B). The EMT switch TE8iresR cell line was partially sensitive to capivasertib or PP242 (consistent with the maintenance of AKT/mTOR signalling). However, neither drug increased TE8iresR sensitivity to gefitinib (Figure S11B).

**Fig. 6:**
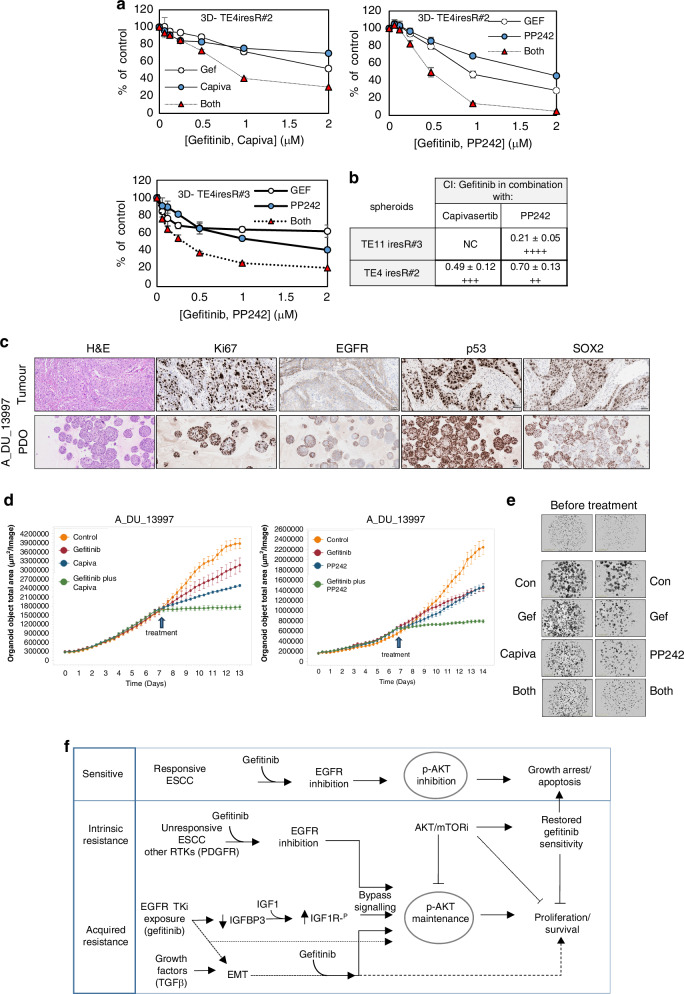
The potential for targeting EGFR and the AKT/mTOR resistance hub. **a** Representative spheroid assays of TE4iresR#2 and TE11iresR#3 gefitinib-resistant cells treated for five days with inhibitors alone or in combination. Data shown are CellTitre-glo® viability counts as a percentage of control (mean ± sd of triplicate wells). **b** Summary table of mean ED75 combination indices (CI) of gefitinib combined with capivasertib (AKTi) or PP242 (mTORi) from at least three independent assays (examples shown in (**a**). CI < 1 = synergy, >1= antagonism; the degree of synergy is given as: ++++ = strong synergism, +++ = synergism, ++ = moderate synergism. NC = could not be calculated. **c** IHC of FFPE patient-derived organoid A_DU_13997 in comparison with diagnostic tumour FFPE tissue. **d** PDO growth analysed by IncuCyte multi-organoid assay (data shown are mean ± sem total organoid area). The arrow indicates the start of drug treatment after organoid formation. **e** Representative multi-organoid images from the experiments described in (**d**). **f** Model of AKT/mTOR signalling as a targetable hub for multiple modes of resistance to EGFR inhibitors. Intrinsic and acquired resistance to gefitinib is associated AKT/mTOR signalling, often through alternative RTKs, for example, PDGFRβ dependency and IGF1R activation. Targeting both EGFR and the downstream AKT/mTOR effectors of alternative RTK signalling restores sensitivity. AKT/mTOR, therefore, is a targetable hub at the centre of multiple different modes of resistance to EGFR inhibitors.

In 3D assays, TE4iresR#2 cells formed spheroids more readily than parental TE-4 cells, so we could assess the effect of therapy on spheroids in all gefitinib-resistant lines. The effects of capivasertib or everolimus were minimal; however, both active site mTOR inhibitors, PP242 and INK128, reduced spheroid growth (Figure S12) as monotherapies, and both capivasertib and PP242 worked synergistically with gefitinib; representative dose-response curves are shown in Fig. 6a, and CI values in Fig. 6b. TE8iresR cells were unaffected by the treatment, therefore, the combination may be more beneficial in gefitinib-resistant cells with activation of the IGF1R pathway rather than in cells with an EMT phenotype.

Patient-derived tumouroids/organoids more closely resemble the phenotypes of the tissue of origin; we therefore generated a new EGFR-expressing PDO (Fig. 6c) and assessed its response to the drug combinations. Gefitinib combined with capivasertib or PP242 was highly effective in preventing PDO growth (Fig. 6d, e), and we observed the same enhanced inhibition of mTOR signalling (Figure S13) as described in Fig. 5f.

These data demonstrate that the AKT/mTOR pathway forms a convergence point for multiple modes of resistance to EGFR inhibitors in ESCC (Fig. 6f). AKTi and/or mTORi as single agents can inhibit ESCC cell migration, anchorage-dependent and anchorage-independent growth, act synergistically with gefitinib, and may restore sensitivity following the development of gefitinib resistance.

## Discussion

The effective clinical use of EGFR inhibitors for oesophageal cancer patients has been hampered by a lack of optimal patient stratification in trials, imperfect predictive biomarkers, non-responders to treatment and the development of drug resistance in all patients who had initially derived benefit from treatment [43].

In EGFR-driven ESCC, we propose that combined EGFR and AKT/mTOR pathway inhibitors may improve drug efficacy in the clinic regardless of the diversity of upstream resistance pathways involved and, given the synergistic responses seen, should enable dose-reduction strategies to limit observed toxicity. The clinical relevance of these findings is increased by observations, supported by NSCLC clinical trials, suggesting that EGFR-driven ESCCs are unlikely to respond well, or durably, to immune checkpoint inhibitors used in adjuvant and palliative care settings in ESCC, and so require alternate treatment strategies. The association of EGFR with the concerning phenotype of CPI-induced hyper-progression characterised by accelerated tumour growth and clinical deterioration [21, 44] indicates further caution for the use of CPIs in EGFR-driven tumours, including ESCCs [19, 20].

### Bypass signalling as a mechanism for maintenance of p-AKT in ESCC

We saw increased signalling via PDGFRβ in intrinsic resistance and IGF1R in acquired resistance. IGFBP3 in cancer has been studied predominantly in lung cancer but its role is still unclear [45]. In EGFR-dysregulated ESCC, we show reduced IGFBP3 with activation of IGF1R and functional effects of IGFBP3 on EGFR TKi responses which support further investigation of IGFBP3/pIGF1R levels in tissue samples from ESCC patients receiving EGFR-targeted therapy. The involvement of IGF1R signalling in ESCC is given greater significance by the observation that it also has a role in resistance to conventional chemotherapeutics, shown here in the GO2 trial and also previously with cisplatin, 5-fluorouracil and camptothecin treatments [46, 47]. Thus, targeting IGF1R or downstream effectors like AKT/mTOR could enhance the efficacy of multiple different treatment strategies.

IGF1R protein expression is associated with tumour progression [47], while co-overexpression of EGFR and IGF1R has been independently confirmed in 64% (48/75) of ESCC patients [48]. As monotherapies, IGFR1 inhibitors have not yet proved clinically useful because of poor toxicity profiles and limited responses. In the shorter term, therefore, the combination of EGFR inhibitors with inhibitors of the downstream effectors AKT/mTOR, which are more advanced in drug development programmes, may be a more suitable choice for development in the clinic.

### Utility of targeting PI3K/AKT/mTOR pathway drug resistance hubs in delivering EGFR targeted therapy

Our data shows that the synergistic targeting of AKT/mTOR and EGFR could improve efficacy but is also associated with elevated toxicity in our xenograft model, requiring dose reduction and scheduling strategies. Other studies using less selective PI3K/AKT inhibitors (wortmannin and LY294002) than those used here have shown efficacy in animal models of oesophageal cancer in combination with standard chemotherapies 5-Fu and cisplatin [36]. As biomarkers, in oesophageal cancers, previous studies have detected 19/29 (65.5%) PIK3R1-positive cancers [49] and considerable variation in constitutively activated AKT (25–90%) in cancer tissues compared with normal tissue [36, 50, 51]. This variation could be attributed to the high sensitivity of immunoreactive p-AKT to fixation methods and antibody selection. Nevertheless, elevated p-AKT and downstream mTOR targets appear associated with poor prognosis [50] and are constitutively activated in lymph node metastases [52]. Likewise, despite a considerable degree of heterogeneity in IHC-positive cases across the different studies analysed, a meta-analysis of 915 ESCC patients showed that mTOR and p-mTOR and high expression of the mTOR targets p-p70S6K and p-4E-BP1 are significantly correlated with unfavourable disease progression and survival [53]. The PI3K/AKT/mTOR pathway is therefore associated with poor prognosis and resistance to conventional chemotherapy and is a valid target for chemotherapy in ESCC [54]. Our data further indicate that intrinsic and acquired gefitinib resistance correlates with the maintenance of basal AKT activation in the face of EGFR inhibition rather than with significantly higher levels of p-AKT per se. Retrospective patient studies suggest otherwise [55], but because of the instability of pAKT in fixed tumour tissue, prospective studies investigating pAKT activity upon treatment with EGFR inhibitors would be useful.

It is possible that targeting mTOR, instead of PI3K or AKT, may be highly effective because it would negate the effects of AKT feedback and PI3K-independent pathways of mTOR activation. This was supported by the effective PDO growth inhibition seen using gefitinib plus an active site mTOR inhibitor. Further work is now required to stratify patients likely to benefit from either monotherapy or combination therapy, and to consider further dose reduction to limit toxicity, which enhanced efficacy of the combination should enable. It is possible that PDGFRβ high expressors could be intrinsically resistant to gefitinib despite having the EGFR CNG biomarker, in which case, AKT/mTOR inhibitors may be better treatment options. However, EGFR TKi-resistant cells with an EMT phenotype remain a challenge, being also more resistant to AKT/mTOR inhibitors in our experiments. In vivo longitudinal studies to determine whether co-administration of EGFR inhibitors with AKT/mTOR inhibitors prevents the development of acquired resistance and resistant cell growth are now also required and would be best performed in PDO xenograft model systems. In addition, immune cell/ESCC PDO co-culture and in vivo models would be valuable resources to improve the efficacy of immune checkpoint inhibitors in EGFR-positive tumours. These concepts warrant further investigation in ESCC, particularly considering the poor outcome of these patients and the unsuitability of EGFR-positive tumours for treatment with immune checkpoint inhibitors.

## Supplementary information

Supplementary Data_Revised

Supplementary Methods_Revised

## Data Availability

Data used in this study are available via the following links: Gene Expression Omnibus (GEO; ncbi.nlm.nih.gov/geo/); The Cancer Genome Atlas (TCGA; cancergenome.nih.gov); TRGAted(https://nborcherding.shinyapps.io/TRGAted/); OSescc (bioinfo.henu.edu.cn/DBList.jsp).
