# Supplementary material for: AKT/mTOR as a targetable hub to overcome multimodal resistance to EGFR inhibitors in oesophageal squamous cell carcinoma
Source: Br J Cancer. 2025 Jul 4;133(5):709–22. doi: 10.1038/s41416-025-03093-3 (PMC12405532; doi:10.1038/s41416-025-03093-3)
Supplement: Supplementary file 1 — Supplementary Data_Revised [file 41416_2025_3093_MOESM1_ESM.pdf]

A

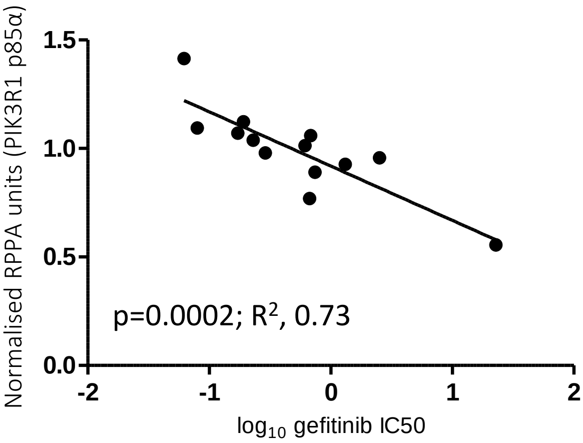

B

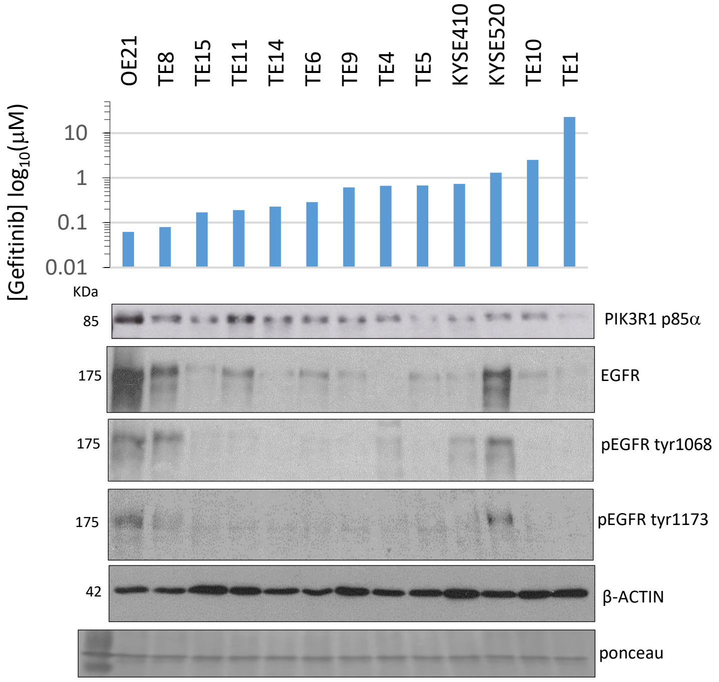

Figure S1. Inverse correlation of PIK3R1 p85α with sensitivity to gefitinib.

(A) GraphPad linear regression analysis of $\log_{10}$ gefitinib IC50 (determined in Figures 1A and 1B) and PIK3R1 p85α protein detected by reverse phase protein array (RPPA) of the ESCC cell line panel. **(B)** Bar chart of $\log_{10}$ gefitinib IC50 of ESCC cell line panel in order of sensitivity. ESCC cell lysates generated independently of the samples analysed by RPPA were analysed by SDS-PAGE and western blotting for the proteins indicated. A β-ACTIN western blot and a representative ponceau-stained nitrocellulose membrane are included as sample integrity controls.

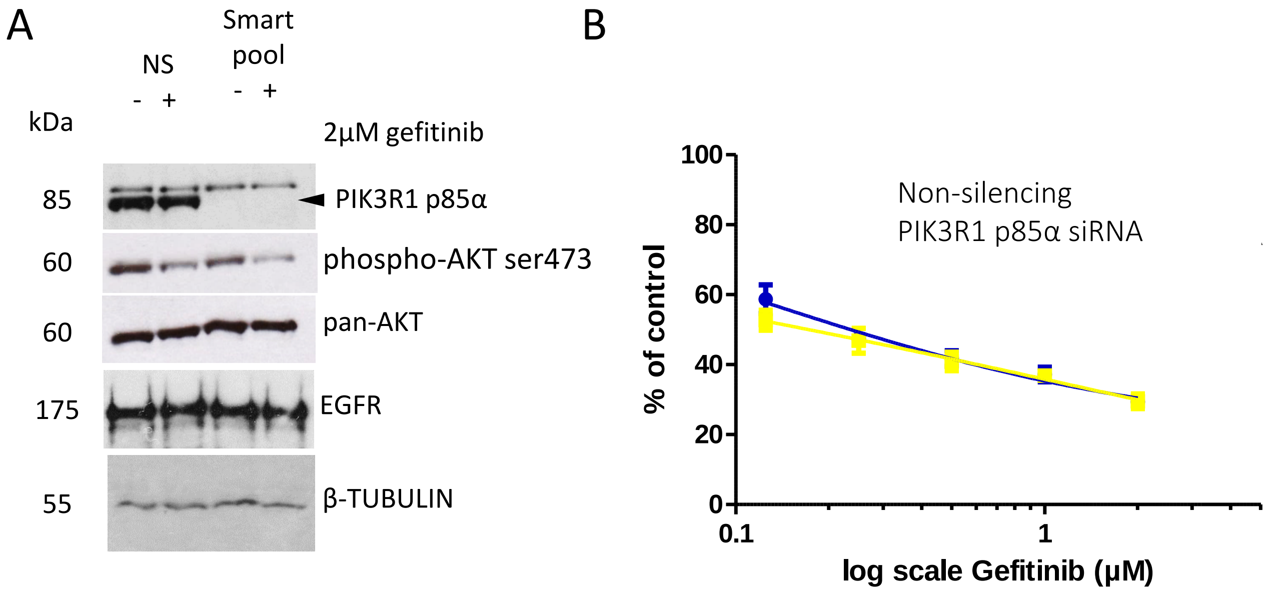

Figure S2 Investigation of resistance to gefitinib in ESCC: (A,B) Transient PI3KR1 p85α knockdown does not induce gefitinib resistance. (B) *PIK3R1 p85α* knockdown in TE-11 cells using smartpool siRNA compared with control non-silencing (NS) siRNA. Following knockdown, cells were seeded into 6–well plates and treated with 2μM gefitinib for 5 hours. Cell lysates were analysed by SDS-PAGE and western blot. A sample integrity blot of β-TUBULIN is included. (C) Gefitinib dose-response analysis of control and *PIK3R1* knockdown TE-11 cells analysed by CellTitre-Glo. Data shown is the mean and sem of independent siRNA transfections [NS, n=6; PIK3R1, n=5] from at least two independent assays.

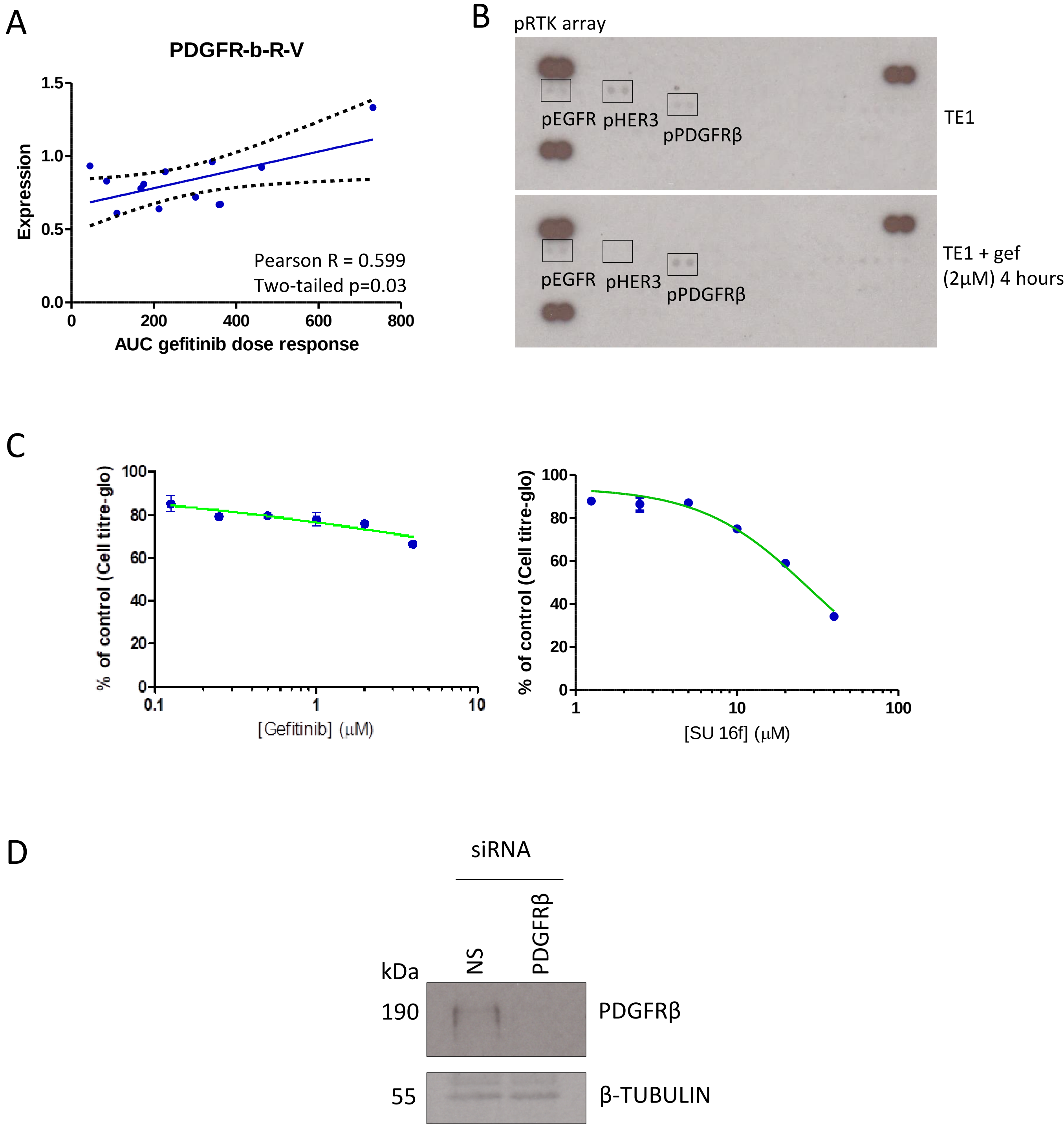

Figure S3. PDGFRβ expression involvement in gefitinib resistance. (A) Linear regression analyses performed in GraphPad using gefitinib dose-response curve (determined in Figure 1A and 1B) and PDGFR protein expression detected by a reverse phase protein array. **(B)** Proteomic profiling by phospho-RTK array (R and D systems) of lysates from untreated and gefitinib-treated (4 hours, 2µM) intrinsically resistant TE1 cells **(C)** Cell proliferation of TE1 cells treated with the EGFR inhibitor gefitinib or the PDGFRβ inhibitor SU16F determined by CellTitre glo®. The mean ± sd cell viability is presented as a percentage of the untreated controls (set at 100%) from independent assays (n=3). Curve fits were analysed in GraphPad prism. **(D)** Western blot showing siRNA knockdown of PDGFRβ in TE-1 cells with a sample integrity western for β-TUBULIN.

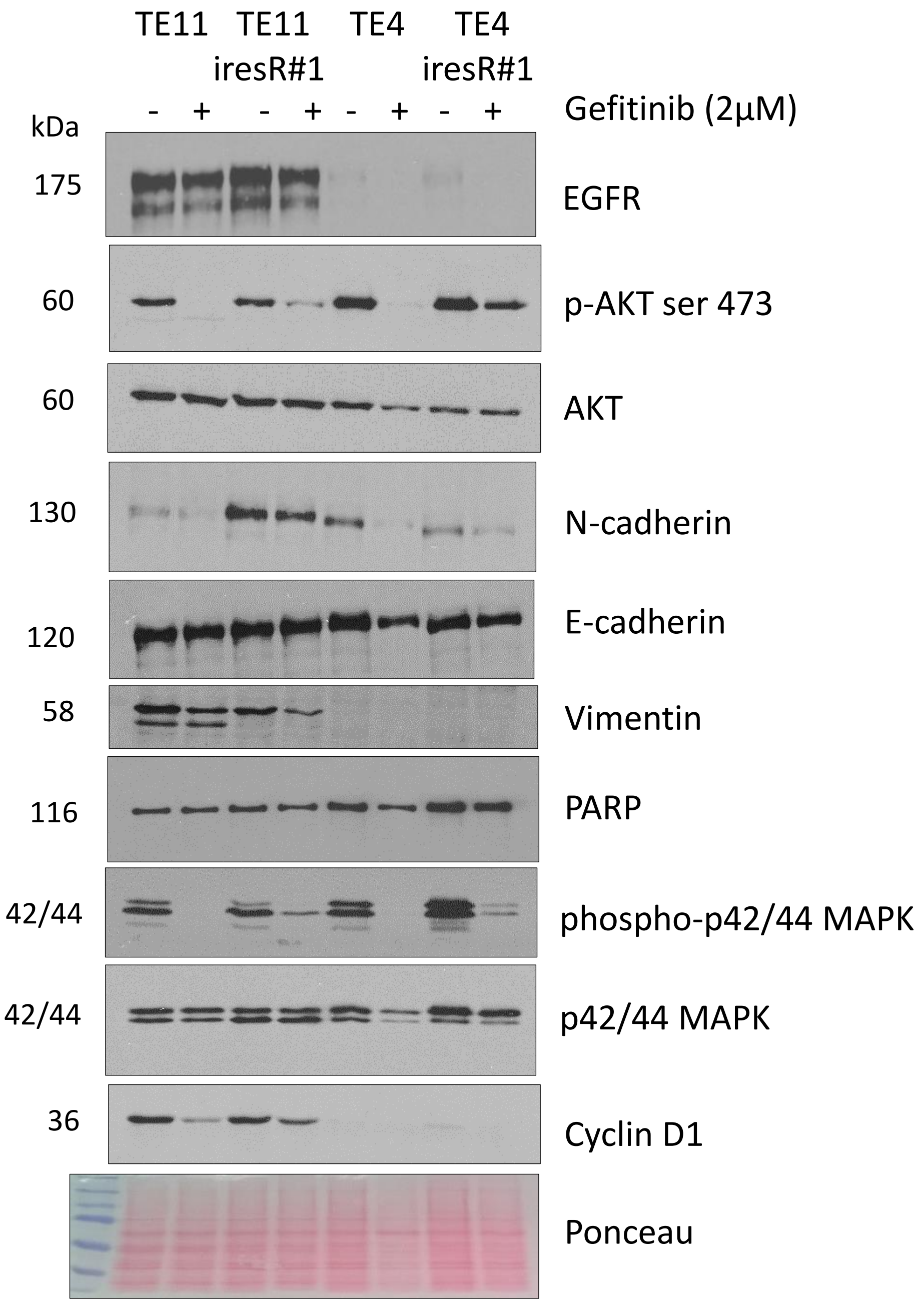

Figure S4. Maintenance of p-AKT correlates with drug resistance in TE11 and TE4 gefitinib-resistant cell lines. SDS-PAGE western blot analysis of protein expression in lysates from ESCC-resistant cell lines compared to control cell lines following 24-hour treatment with and without 2μM gefitinib. Shown are representative gels and a sample integrity ponceau stained nitrocellulose membrane.

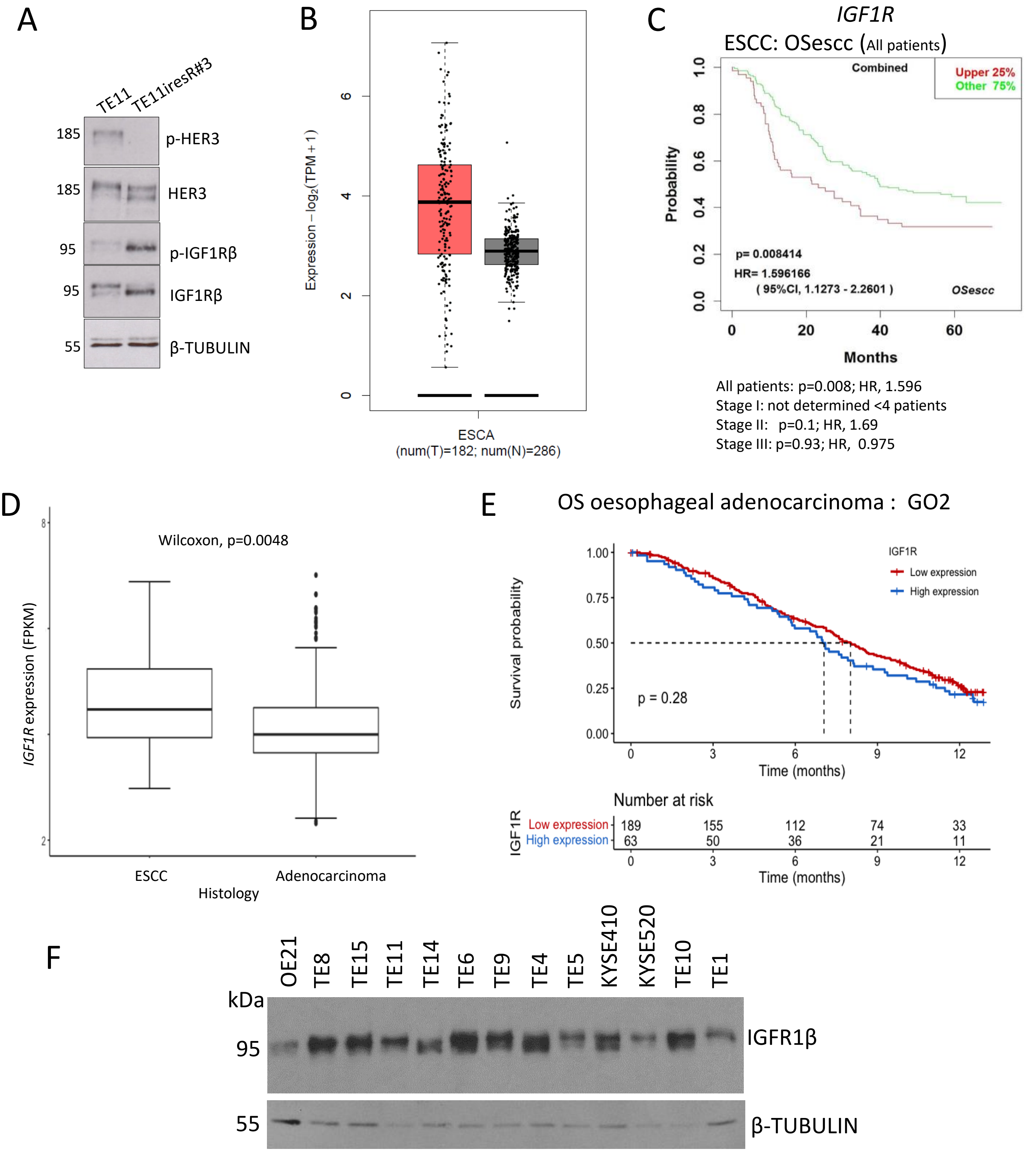

Figure S5. (A) pRTK array lysates were analysed by SDS-PAGE and western blotting. β-TUBULIN is shown as a sample integrity control. (B) *IGF1R* RNA expression in oesophageal cancer tissue compared to normal tissue. GEPIA2 analysed data is from TCGA tumour (n=182) (red box plot) and matched samples plus GTEx normal tissue (n=286) (grey box plot), p-value cut-off 0.01. (C) Overall survival from combined TCGA + GSE52635 RNAseq data from ESCC patients (n=264) according to *IGF1R* expression. High expression defined as top quartile of expressors. Low expression defined as bottom 75% of expressors by OSescc analysis. (D) A comparison of *IGF1R* RNA expression in GO2 trial cohort gastroesophageal cancer tissues with either ESCC (n=32) or EAC (n=252) histology. (E) Overall survival in the GO2 trial GOA population according to *IGF1R* RNA expression. High expression defined as top quartile of expressors. Low expression defined as bottom 75% of expressors. (F) IGFR1 protein is expressed in ESCC cell lines. SDS-PAGE western blot analysis of IGFR1β expression in lysates from ESCC cell lines with a sample integrity blot for β-tubulin.

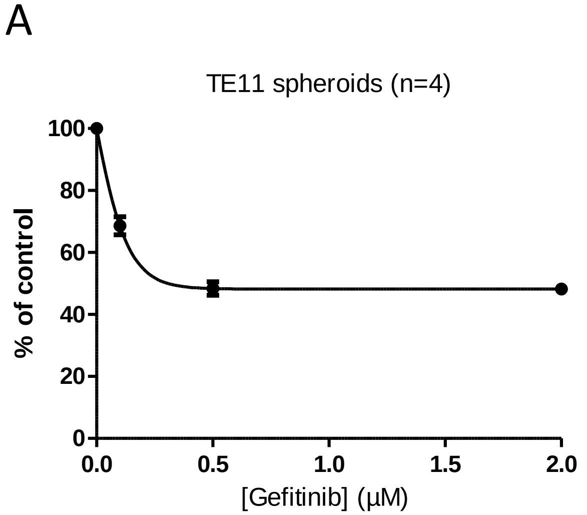

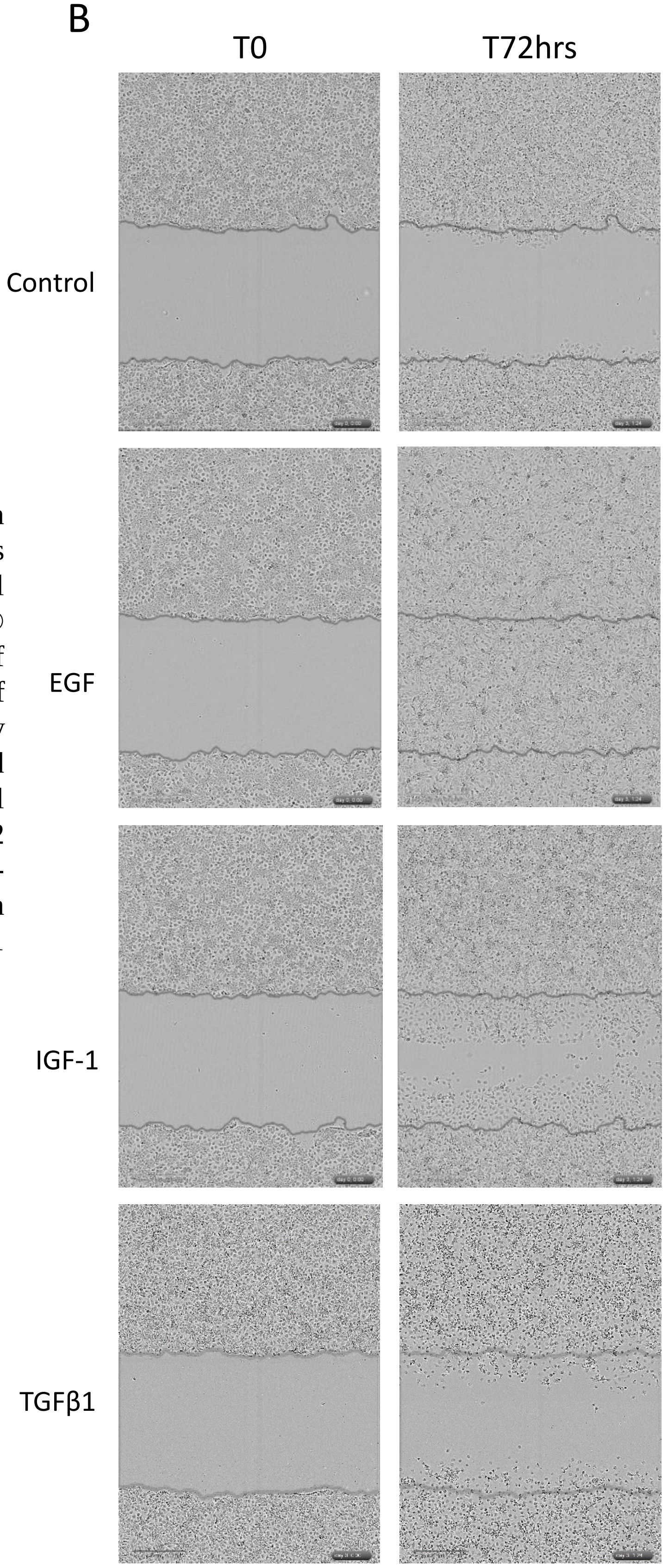

Figure S6. (A) TE-11 cells were plated in anchorage-independent 3D- spheroid assays and treated with gefitinib for 5 days. Cell viability was assessed by CellTitre-glo® assay and results are expressed as the % of control untreated cells (mean ± s.d) of replicate assays each with 6 wells per assay (n=4). **(B)** TE-11 migration assay analysed by IncuCyte software showing the initial wound mask at T0 (left panels) and after 72 hours cell migration (right panels) in serum-free medium (control) or serum-free medium supplemented with EGF (5ng/mL). IGF-1 (100ng/mL) or TGFβ1 (5ng/mL).

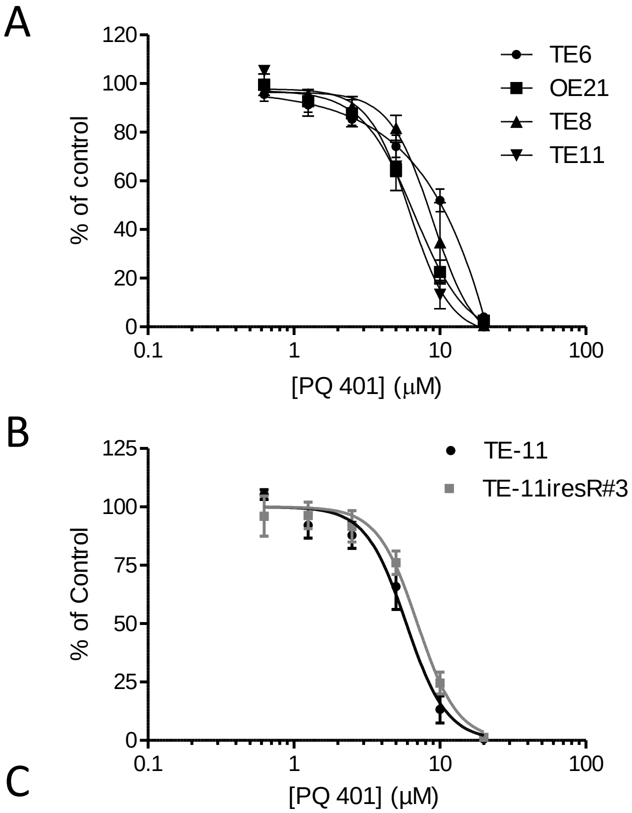

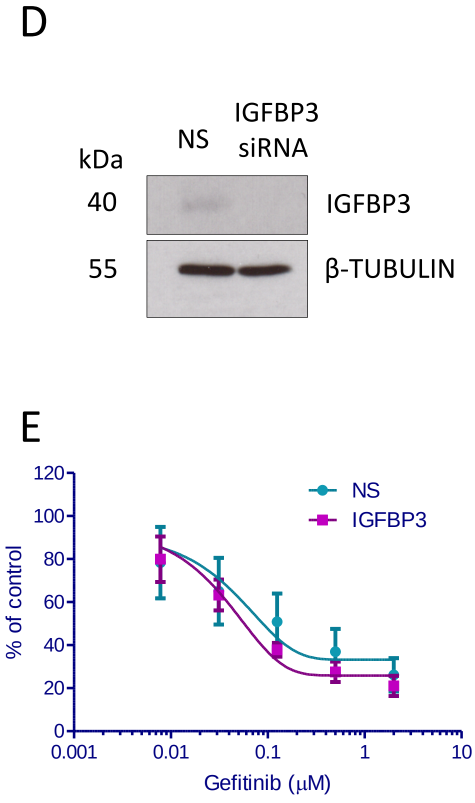

C

-	Combination Indices (mean ± sd, n=3)		
**Cell line**	ED50	ED75	ED90
**TE-11**	0.97±0.25<br>±	0.45±0.05<br>+++	0.51±0.05<br>+++
**TE-11iresR#3**	1.23±0.30<br>– –	1.02±0.15<br>±	0.87±0.06<br>+

Figure S7. (A, B) Dose response of the IGF1R inhibitor PQ401 in ESCC cells **(A)** and TE-11 parental and TE11iresR#3 cells **(B)**. Data shown is the mean ± sd non-linear curve fit determined in Graphpad Prism from independent CellTitre-glo® assays (n=3). **(C)** Combination indices from gefitinib and PQ401 drug combination studies in TE-11 cells. CI values were determined by CalcuSyn software and are shown as the mean ± sd CI from replicate experiments (n=3). CI <1 = synergy, >1= antagonism; recommended symbols for the degree of synergy is given as: +++++ = very strong synergism ++++ = strong synergism, +++ = synergism, ++ = moderate synergism, + = slight synergism , ± = nearly additive, - = slight antagonism, -- = moderate antagonism, --- = antagonism, ---- = strong antagonism, ----- = very strong antagonism. (D) siRNA knockdown of IGFBP3 in TE11 cells NS = non-silencing siRNA. β-TUBULIN western is included as a sample integrity control. (E) CellTitre-glo® cell viability gefitinib dose response curves in non-silencing control and IGFBP3 knockdown TE11 cells (n=3). Data shown is the mean ± sd non-linear curve fit determined in Graphpad Prism from independent assays (n=3)

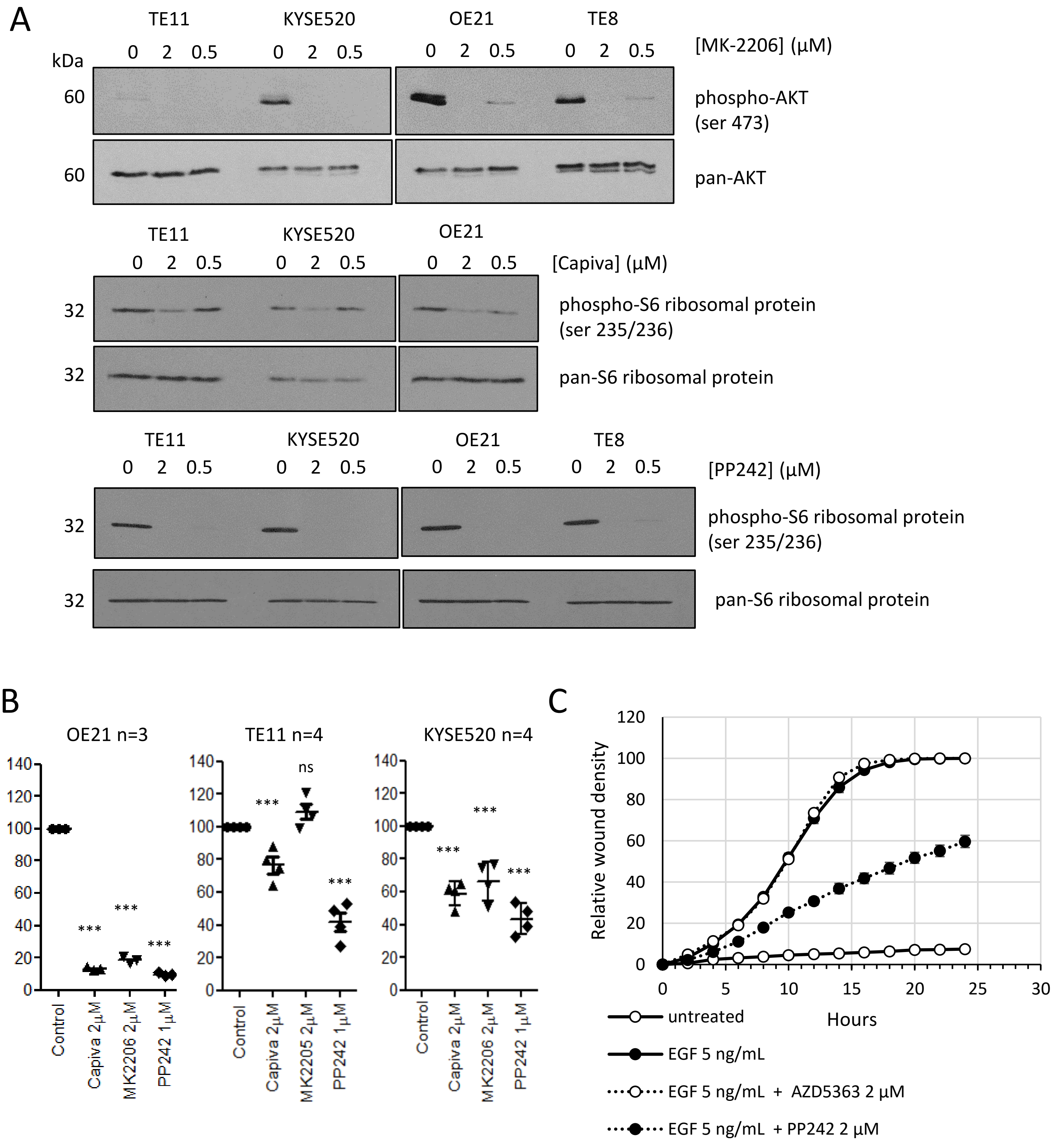

Figure S8 (A) Titration of the AKT inhibitors MK2206 and Capivasertib (Capiva), and the mTORC1/2 inhibitor PP242 on intrinsically gefitinib sensitive (TE-11, TE-8 and OE21) and resistant (KYSE-520) ESCC cell lines. Western blot analysis of equal amounts of protein lysate was carried out using pAKT ser473 for MK2206, and AKT substrates S6 ribosomal protein for Capiva and PP242. (B) Quantification of crystal violet stained colony formation assays following 1% SDS solubilisation. OD readings were taken from independent experiments (OE21, n=3; TE-11, n=4; KYSE-520, n=4) with at least 3 replicate wells in each experiment. Statistics were performed by ANOVA and post-test Dunnett's correction. **(C)** Effect of Capiva and PP242 on EGF-induced TE-11 cell migration. TE-11 cells were serum starved for 48 hours prior to wounding and treatment with drugs in 0% serum media. Wound closure was monitored by real time IncuCyte Zoom imaging and results are displayed as mean relative wound density ± sem (100% = full wound closure).

A

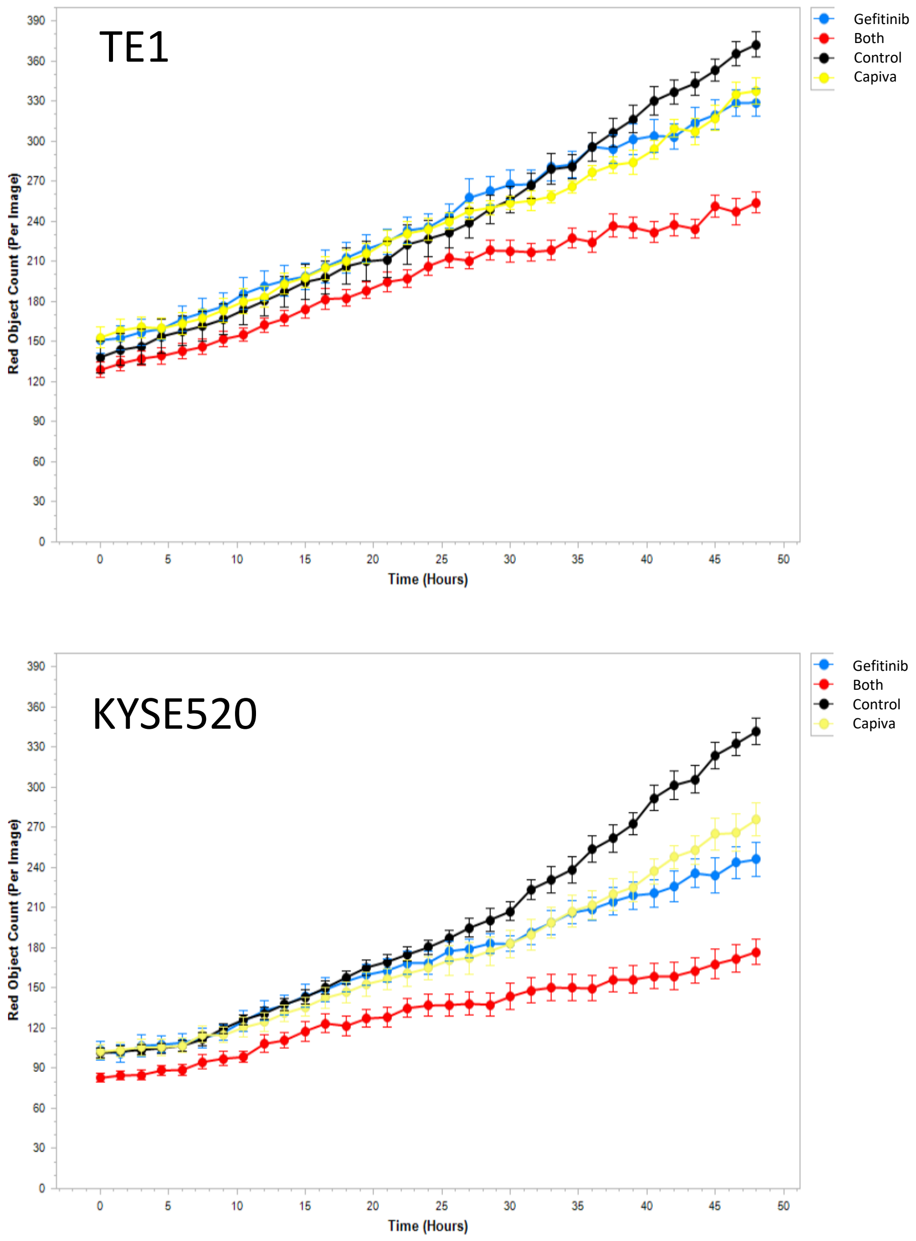

Figure S9. TE-1 and KYSE-520 intrinsically resistant cells were labelled with a red fluorescent nuclear dye and treated with gefitinib (2µM), capivasertib (0.5µM) or both drugs for 48 hours. Cell number (red object/image) was monitored by real-time imaging every 90 minutes on an IncuCyte S3 live cell imager.

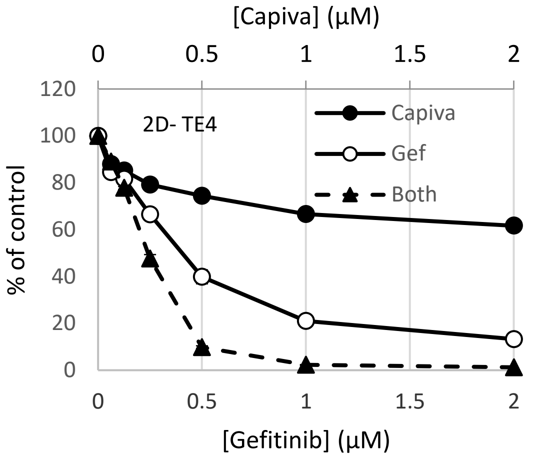

Figure S10. Combination drug treatment of TE4 cells used in the murine CDX experiment

	Placebo (N=7)	Capiva (high) (N=8)	Combination (N=13)	Gefitinib (high) (N=6)	Total (N=34)
**Cause of death**					
Censored	0 (0%)	0 (0%)	1 (7.7%)	0 (0%)	1 (2.9%)
Tumour to size	4 (57.1%)	2 (25.0%)	0 (0%)	6 (100%)	12 (35.3%)
Weight loss	0 (0%)	0 (0%)	2 (15.4%)	0 (0%)	2 (5.9%)
Ulceration	3 (42.9%)	6 (75.0%)	10 (76.9%)	0 (0%)	19 (55.9%)

Table S2 Cause of death of CDX mice following drug combination treatment

**Characteristic**	**HR**[1]	**95% CI**[1]	**p-value**
Placebo	—	—	
Capiva (high)	0.54	0.19, 1.55	0.3
Capiva (low)	0.37	0.12, 1.08	0.068
Combination	**0.33**	**0.12, 0.88**	**0.028**
Gefitinib (high)	0.51	0.17, 1.52	0.2
Gefitinib (low)	0.36	0.12, 1.12	0.078

[1] HR = Hazard Ratio, CI = Confidence Interval

Table S3 Cox regression analysis of CDX murine combination drug treatment.

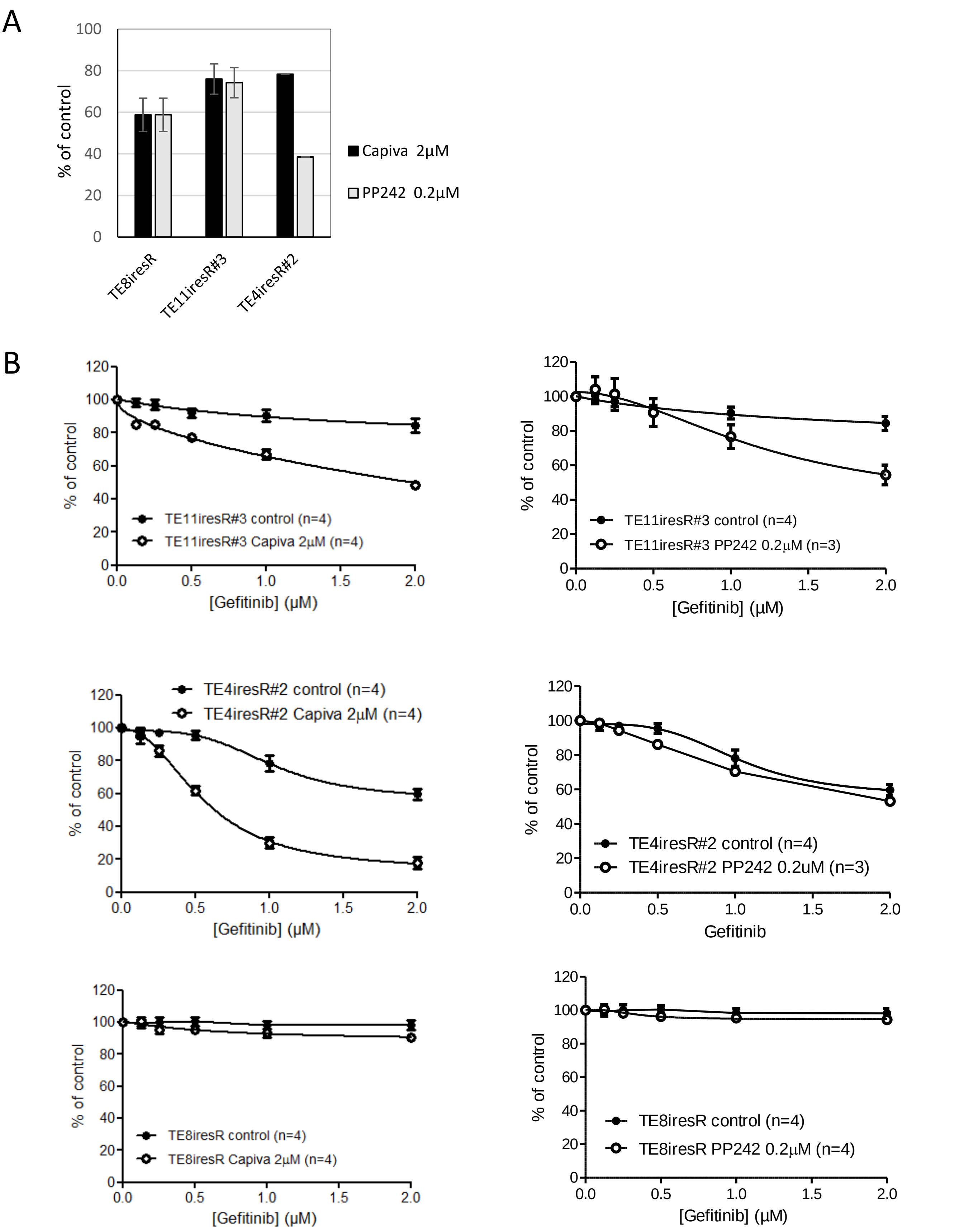

Figure S11. (A) Effect of single dose of capivasertib (Capiva) (2µM), and the mTORC1/2 inhibitor PP242 (0.2µM) on gefitinib acquired-resistant ESCC cell line proliferation in adherent 2D assays. **(B)** Gefitinb-resistant cells were treated with a single dose of capiva (2µM), and PP242 (0.2µM) as shown in (A) in combination with gefitinib. Cell proliferation was determined by CellTitre-glo™ and results are expressed as the mean ± sd percent of their own (no gefitinib) controls from independent replicate assays (n).

Spheroid assays

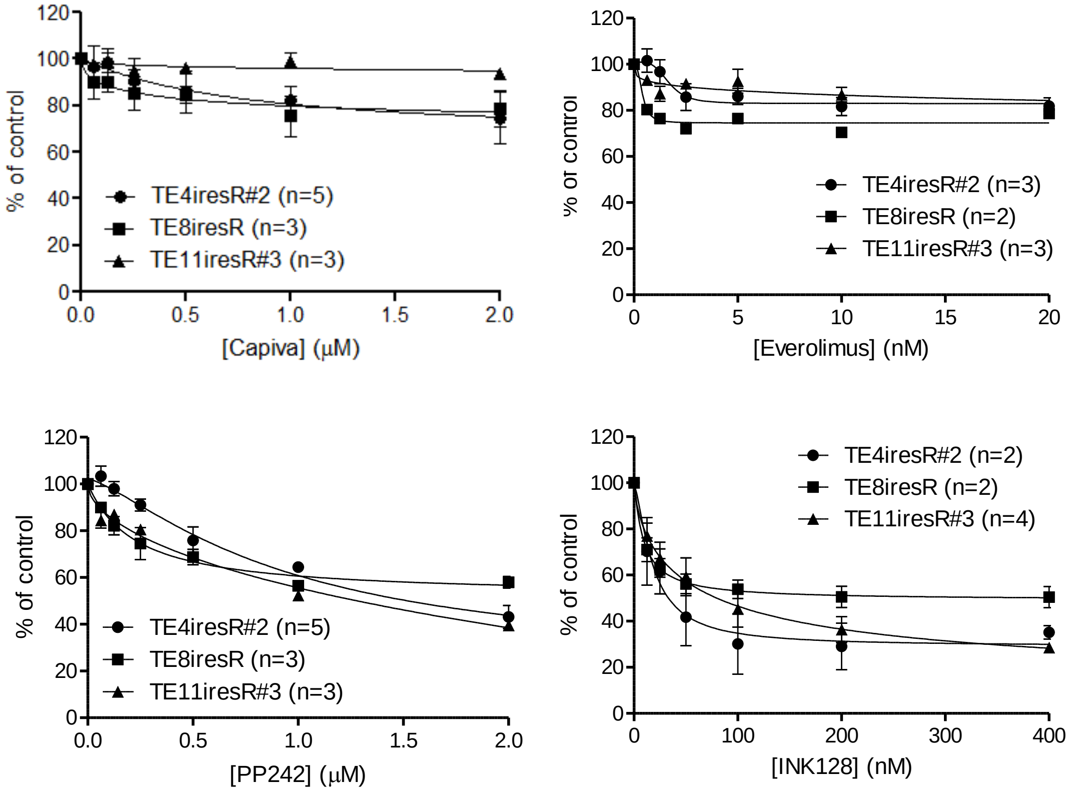

Figure S12 AKTi and mTORi monotherapy treatment of acquired gefitinib-resistant cell lines grown as 3D spheroids. Spheroids were treated for 5 days with a titration of capivasertib, PP242, everolimus or INK128 before analysis by CellTitre-glo® assay. Data shown is the non-linear curve fit (mean % of control ± sd) determined in GraphPad prism from independent assays (n on graph).

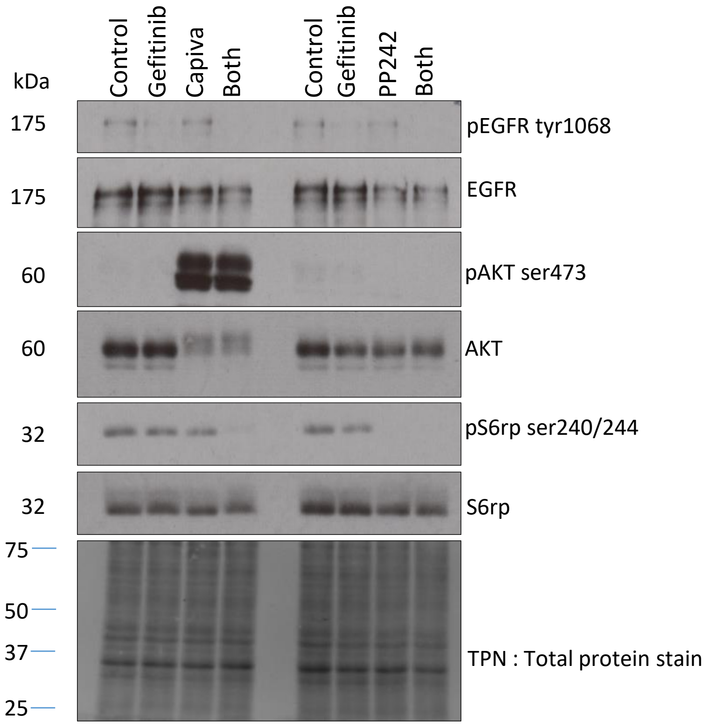

	Control	Gefitinib	Capiva	Both	Control	Gefitinib	PP242	Both
pEGFR	100	19	105	4.2	100	13	64	6.6
EGFR	100	112	72	48	100	98	52	45
pS6rp	100	76	50	6	100	64	1.5	0
S6rp	100	86	87	75	100	91	67	68

TPN normalised expression as % of control

Figure S13. Combination treatment of A_DU_13997 PDO. Organoids grown in Matrigel and PDO growth media were allowed to establish and then treated with drug for 24 hours before protein extraction and analysis by western blotting. A representative membrane stained with TPN reagent is shown, protein expression was analysed by Image Studio and was normalised according to the TPN total protein stain measured using iBright™ analysis software. Expression is shown as a percent of the untreated sample.
