# Supplementary material for: AKT/mTOR as a targetable hub to overcome multimodal resistance to EGFR inhibitors in oesophageal squamous cell carcinoma
Source: Br J Cancer. 2025 Jul 4;133(5):709–22. doi: 10.1038/s41416-025-03093-3 (PMC12405532; doi:10.1038/s41416-025-03093-3)
Supplement: Supplementary file 2 — Supplementary Methods_Revised [file 41416_2025_3093_MOESM2_ESM.pdf]

Supplementary Methods

Cell lines

The cell lines used in the study were obtained from the Cell Resource Center for Biomedical Research, Institute of Development, Aging and Cancer, Tohoku University, Japan: TE-1 (RRID:CVCL_1759), TE-4 (RRID:CVCL_3337), TE-5 (RRID:CVCL_1764), TE-6 (RRID:CVCL_1765), TE-8 (RRID:CVCL_1766), TE-9 (RRID:CVCL_1767), TE-10 (RRID:CVCL_1760), TE-11 (RRID:CVCL_1761), TE-14 (RRID:CVCL_3336), TE-15 (RRID:CVCL_1763), KYSE-520 (RRID:CVCL_1355), KYSE-410 (RRID:CVCL_1352) and OE21 (RRID:CVCL_2661). They were maintained in Roswell Park Memorial Institute RPMI medium 1640 supplemented with L-glutamine (GIBCO) and 10% foetal bovine serum (FBS) (GIBCO). The cell lines tested negative for mycoplasma by the in-house testing facility (Mycoalert) and were independently authenticated by STR profiling (NorthGene Ltd, Newcastle UK).

Reagents and antibodies

Stock inhibitor solutions were prepared as follows:

EGFR inhibitor gefitinib (Iressa) (Tocris), 20mM in DMSO. The AKT inhibitor (AKTi) Capivasertib (Truqap™, AZD5363) (34) (Selleckchem), was prepared in 20mM in DMSO. Capivasertib inhibits the active site of all AKT isoforms AKT1, AKT2 and AKT3. MK-2206 was prepared in 20mM in DMSO. MK-2206 is an allosteric non-ATP competitive inhibitor of all AKT isoforms AKT1, AKT2 and AKT3. The inhibitor PP242 was prepared at 10mM in DMSO. PP242 is an active site dual inhibitor of mTORC1 and mTORC2. The active site dual mTORC1/2 inhibitor INK-128 (also called MLN0128, TAK228, sapanisertib) (APExBIO) was prepared at 1mM in DMSO. The IGF1R inhibitor PQ401 (Tocris) was prepared at 20mM in

DMSO. Everolimus is a selective inhibitor of mTORC1 through binding of FKBP12. Everolimus (Afinitor®) was prepared at1mM in DMSO.

Recombinant proteins

Recombinant proteins from Peprotech were prepared as follows: human (rh) rhTGFβ1 was reconstituted in 1mg/mL bovine serum albumin (BSA)/4mM HCL at 5μg/mL, aliquoted and stored at -80ºC and was used at a final concentration of 5ng/mL (1); rhIGF-1 was reconstituted at 50μg/mL in 0.1% BSA and stored at -80ºC; rhEGF was reconstituted at 200μg/mL in 1mg/mL BSA and stored at -80ºC. rhIGFBP3 was reconstituted in 1mg/mL BSA at 500 μg/mL aliquoted and stored at -80ºC and used at concentrations of 2.5μg/mL corresponding to normal serum concentrations (2).

Antibodies and western blotting

Antibodies used in western blotting experiments were purchased from Cell Signalling Technology unless stated otherwise and are as follows: rabbit mAb anti-phospho AKT Thr 308 (244F9) (RRID:AB_331163), rabbit polyclonal anti-phospho AKT ser 473 (RRID:AB_329825), rabbit mAb anti-phospho-EGFR Y1173 (53A5) (RRID:AB_331795), rabbit mAb anti-EGFR (D38B1) (RRID:AB_111290260), rabbit mAb anti-phospho-EGFR Y1068 (D7A5) (RRID:AB_2096270), rabbit mAb anti-PDGFRβ (28E1) (RRID:AB_2924258), rabbit mAb anti-IGF1Rβ (D406W) (RRID:AB_2665558), rabbit mAb anti-phospho-IGF1Rβ (Tyr1135) (DA7A8) (RRID:AB_10548764), rabbit mAb anti-HER3/ErbB3 (D22C5) (RRID:AB_2721919), rabbit mAb anti-phospho-HER3/ErbB3 (Tyr1289) (21D3) (RRID:AB_2099709), mouse anti-S6 ribosomal protein (54D2)

(RRID:AB_2238583), rabbit polyclonal anti-phospho-S6 ribosomal protein (Ser235/236) (RRID:AB_331679), rabbit polyclonal anti-phospho S6 ribosomal protein (Ser 240/244) (RRID:AB_331682), mouse monoclonal anti-GSK3β (3D10) (RRID:AB_10839406), rabbit monoclonal anti-phospho-GSK3β (Ser9) (5B3) (RRID:AB_2115201), mouse mAb anti-IGFBP3 (E-9) (Santa-Cruz biotechnology) (RRID:AB_10988386), mouse mAb anti-β-tubulin (Santa-Cruz biotechnology) (RRID:AB_2288090), mouse mAb anti-N-cadherin (32) (BD Biosciences) (RRID:AB_398236), mouse mAb anti-E-cadherin (610182) (BD Biosciences) (RRID:AB_397581), mouse mAb anti-VIMENTIN (V9) (Sigma) (RRID:AB_477627), rabbit polyclonal anti-PI3K p85alpha n-SH2 domain (Millipore) (RRID:AB_2722790). Secondary antibodies were polyclonal goat anti-rabbit Ig-HRP conjugate (Dako) and goat anti-mouse Ig-HRP conjugate (Dako).

Monitoring and analysis of cell proliferation and colony formation

Cell-titre-Glo cell viability assays.

1000 or 5000 (TE4 and TE6 only) viable cells/well were seeded overnight in 96-well plates. Cells were then treated with either solvent control or 2 – 4-fold dilutions of the drug. For drug co-administration studies, drug dilutions used were designed to conform to the requirements outlined for analysis by the Chou-Talalay mathematical model of drug combinations (27), namely, that combination drugs were used at equimolar dilution ratios at predetermined concentrations where they affected cell growth. Proliferation assay endpoints (control wells 80% confluent during log-phase growth) were analysed by CellTiter-*Glo*® Luminescent *Cell* Viability Assay (Promega) according to the manufacturer’s instructions. Data were calculated as a percent of the cell line control (untreated/vehicle-treated cells). Analysis was performed by first determining the mean of any technical replicates within an

assay. Assays were then independently repeated (n) before further analysis and statistical testing unless stated otherwise.

Spheroid assays

Microtitre plates (U-bottomed) were coated with 0.5 % poly (2-hydroxyethyl methacrylate) (poly-HEMA) prepared in 95% ethanol (two applications of 50µL dried at 37ºC) and were stored at 4ºC until required. 1000 cells/100µL growth media were seeded into the coated plates and were centrifuged for 10 mins, 2000 rpm. After overnight incubation, spheroids were treated with 100µL of the appropriate reagents for 5 days before endpoint analysis by CellTitre-glo® assay.

Colony formation assay

Cells were seeded overnight at 1000 cells/well before being treated for 2 weeks with appropriate drugs. Colonies were fixed in methanol for 30 minutes, washed, air dried and stained with crystal violet staining solution (0.5%w/v in 20% methanol). Stained colonies were quantified by solubilisation in 1% SDS and the absorbance of the resulting solution was measured at 750nm.

Patient-derived Organoids (PDO)

Patient-derived organoids were prepared from tumour tissue using protocols based on those described previously (3). Briefly tumour tissue was minced in Hanks' balanced salt solution (HBSS) supplemented with dispase (10U/mL), fungizone (amphotericin B) (0.5µg/mL) and Y-27632 10µM. Tissue was placed into a dissociation tube C and incubated on a gentleMACS™ Octo Dissociator (programme 37ºC_h_TDK_3 for 1 hour) (Miltenyi Biotech). The tumour single cell suspension was centrifuged (10s, RT) and the supernatant replaced with 1mL 0.25% trypsin-EDTA, 10 minutes 37ºC. The cell suspension was added to 8mL soybean trypsin

inhibitor (250mg/mL) and centrifuged 1200rpm, 5 minutes. The cell pellet was resuspended in basal media (advanced DMEM/F12, 1x GlutaMax, 10mM HEPES, 1x antibiotic-antimycotic, 5µg/mL gentamicin) and the cell density and viability determined with a trypan blue stain and haemocytometer count. Corning™ Matrigel™ Matrix was thawed on ice and $2x10^4$ cells/50µL seeded in a Matrigel™ dome in pre-warmed 24-well plates. After 30-minutes incubation at 37°C to allow the Matrigel™ to set, 500µL ESCC organoid-specific media was added [basal media supplemented with R-spondin (500ng/mL), noggin (100ng/mL), N-2, B-27, N-acetyl cysteine (NAC, 1mM), Y-27632 (10µM) (for the first 2–3 days during establishment), and gentamicin (10ug/mL)]. Media was replenished every 2–3 days. PDO cells were passaged following monitoring of size and darkness using QC IncuCyte organoid analysis software (usually between 12 and 16 days when organoids reached between 70–150nm size). Generally during passage, Matrigel™ domes from 3 wells containing PDOs were resuspended in cold DPBS, centrifuged (500g, 3 minutes) and the pellet resuspended in 1mL trypsin-EDTA for 10 minutes 37°C. Trypsin was neutralised with soybean trypsin inhibitor and the cells passaged as above.

PDO Immunohistochemistry (IHC)

Sections of formalin-fixed paraffin embedded tumour tissue or PDO blocks (nominally 4 microns thick) were cut onto superfrost® plus slides (VWR International Ltd) and dried for 1 hr at 60 °C. Antigen retrieval and de-paraffinisation was performed using DAKO EnVision™ FLEX Target Retrieval Solution (high pH) buffer (K8004) in a DAKO PT Link for 20 minutes at 97°C. Sections were initially washed in Flex Wash Buffer (K8006) for 5 mins at room temperature and the following steps were performed manually: Flex Peroxidase-Blocking Reagent (SM801) was applied for 5 minutes at room temperature followed by incubation with

primary antibody p53, clone DO-7 (Agilent DAKO M700129-2, 1:100); Ki67, clone MIB-1 (Agilent DAKO M724029-2, 1:100), EGFR, clone D38B1 (Cell Signaling #4267, 1:200) or SOX2, clone D1C7J (Cell Signaling #14962, 1:200) overnight at 4 °C. All antibodies were diluted in DAKO EnVision™ FLEX antibody diluent. The following steps were then performed using a DAKO using a DAKO Link Autostainer. Flex/HRP labelled polymer (SM802) for 20 minutes, Flex DAB+ working solution (SM803) for 2 x 5 minutes, copper sulphate solution for 5 minutes, Flex Haematoxylin for 5 minutes. Between each step, sections were rinsed with Flex Wash Buffer with a final wash in dH_2O. Sections known to stain positively were included in each batch of staining and negative controls were prepared by replacing the primary antibody with DAKO antibody diluent. Slides were manually washed in tap water before being rinsed in graded concentrations of alcohol, with 3 final rinses in Xylene. Glass coverslips were applied and slides scanned.

Reverse Phase Protein array sample preparation

All 13 ESCC cell line replicates were plated in 6 well plates in 2mL media and incubated overnight. The following day media was removed, and the cells were washed twice with ice-cold PBS. 150µL MD Anderson recommended lysis buffer was added to each well, incubating on ice for 20 minutes with gentle agitation every 5 minutes. Cells were scraped and lysates were collected before being centrifuged at 14,000 rpm for 10 minutes at 4°C. The resulting supernatant was collected, and the pellet was discarded. The protein concentration of replicates was determined by Bradford assay and samples were adjusted to a final concentration of 1µg/µL in 4x sample buffer. Samples were subsequently boiled for 5 mins, snap-frozen in liquid nitrogen and stored at -80°C. 60µL of 1µg/µL of triplicate protein samples were analysed at the RPPA core facility at MD Anderson (Houston, Texas). Samples were serially diluted two-fold for 5 dilutions and arrayed on nitro-cellulose coated slides in an 11x11 format.

Samples were probed with antibodies using tyramide-based signal amplification and visualized by DAB colorimetric reaction before being scanned on a flatbed scanner to produce a 16-bit tiff image. Spots from these images were quantified by an Array-Pro Analyser. Relative protein levels for each sample were determined by interpolation of each dilution curve from the "standard curve" (supercurve script function, R) of the slide (per antibody). Log2 data were transformed to linear values for further analysis, in-house.

GO2 trial data analysis

The GO2 trial recruited 559 patients, including both squamous and non-squamous histology. The GO2 trial clinical database is held at the Clinical Trials Research Unit in Leeds Institute of Clinical Trials Research, University of Leeds, Leeds, and all data analysis in this manuscript uses this anonymised dataset. Between November 2014 and January 2018, formalin-fixed paraffin-embedded (FFPE) tumour blocks were collected from 395/559 (70.7%) patients in the trial. RNA was extracted from the GO2 patient FFPE blocks by Almac (Craigavon, Northern Ireland) using the methodology described in Turkington et al. (5). Briefly, H&E sections were assessed for a minimum requirement of 10% viable tumour cell content. Following annotation, 4-5 × 5-µm sections were sectioned and macrodissected for RNA isolation. RNA extraction was carried out using Qiagen RNeasy FFPE extraction kits. RNA sequencing was then performed using the Illumina Tru-Seq® RNA Exome library preparation kit, followed by sequencing on the Illumina NovaSeq with paired-end reads (75 bp) and 50M total reads per sample. Read alignment was performed using StarAlign to the human reference genome GRCh37/hg19. Gene expression data in Fragments Per Kilobase per Million (FPKM) mapped format were generated using Cufflinks for all genes represented in the human reference genome GRCh37 annotation file. Data QC was assessed using housekeeping gene coverage (HK

metric). Only samples with an HK coverage of >/= 2.87 were taken forward for analysis. RNA sequencing data, including from 32 ESCC samples, were analysed for associations between expression groups and progression-free (PF) and overall survival (OS) using Cox proportional hazards regression producing unadjusted and adjusted hazard ratios (HR) and 95% confidence intervals (CIs). All statistical analyses were performed using R statistical software (version 4.0.2).

CDX study

Subcutaneous transplants

All mice were housed in a barrier facility under a 12-hour light-dark cycle with a standard diet and water available ad libitum. $5x10^6$ TE-4 cells were suspended in 100μl of PBS and injected subcutaneously onto the flank of 16-18-week-old female BALB/c Nude mice (Charles River, UK). Tumour measurements were taken three times weekly by staff blinded to the experimental result. The formula [Volume = (length x width^2)/2] was used to calculate the tumour volume. Mice were enrolled onto treatment on day 18 post-transplant, into either placebo (Capivasertib Vehicle + Gefitinib Vehicle), Gefitinib low dose (50mg/kg), Gefitinib high dose (100mg/kg), Capivasertib low dose (85mg/kg), Capivasertib high dose (130mg/kg) or combination (gefitinib low dose + Capivasertib low dose) arms, ensuring similar averaging starting volume across groups.

Animals were monitored and tumour growth measured until humane endpoints were reached (tumours measuring 14-15mm in any dimension, ulceration, 20% weight loss) and were culled by Schedule 1 methods**.**

In vivo drug treatment

Capivasertib (AstraZeneca) was reconstituted at a concentration of 400mg/mL in 100% DMSO. This stock solution was reconstituted to generate a 26mg/mL or 17mg/mL solution in sterile water and 25% W/V Kleptose HPB for high and low doses, respectively. Capivasertib high dose was dosed at 130mg/kg and Capivasertib Low Dose was dosed at 85mg/kg. Capivasertib vehicle was a 10% DMSO, 25% w/v Kleptose HPB (Roquette) solution in sterile water. Capivasertib and Capivasertib vehicle were dosed by oral gavage twice daily, a.m. and p.m., with 4 days on treatment and 3 days off.

Gefitinib was reconstituted in 0.5% Tween-80 (Sigma-Aldrich) in sterile water, to a 20mg/mL or 10mg/mL concentration. Gefitinib High Dose was dosed at 100mg/kg and Gefitinib Low Dose was dosed at 50mg/kg. Gefitinib vehicle was 0.5% Tween-80 in sterile water. Gefitinib and Gefitinib Vehicle were dosed once daily by oral gavage at least 2 hours after and 2 hours before Capivasertib dosing, with 4 days on treatment and 3 days off.

References

1. Spender LC, Ferguson GJ, Liu S, Cui C, Girotti MR, Sibbet G, et al. Mutational activation of BRAF confers sensitivity to transforming growth factor beta inhibitors in human cancer cells. Oncotarget. 2016;7(50):81995-2012.

2. Hoyo C, Grubber J, Demark-Wahnefried W, Lobaugh B, Jeffreys AS, Grambow SC, et al. Predictors of variation in serum IGF1 and IGFBP3 levels in healthy African American and white men. J Natl Med Assoc. 2009;101(7):711-6.

3. Karakasheva TA, Kijima T, Shimonosono M, Maekawa H, Sahu V, Gabre JT, et al. Generation and Characterization of Patient-Derived Head and Neck, Oral, and Esophageal Cancer Organoids. Curr Protoc Stem Cell Biol. 2020;53(1):e109.

4 Turkington RC, Knight LA, Blayney JK, Secrier M, Douglas R, Parkes EE, et al. Immune activation by DNA damage predicts response to chemotherapy and survival in oesophageal adenocarcinoma. Gut. 2019;68(11):1918-27.
